# High-affinity five/six-letter DNA aptamers with superior specificity enabling the detection of dengue NS1 protein variants beyond the serotype identification

**DOI:** 10.1093/nar/gkab515

**Published:** 2021-06-25

**Authors:** Ken-ichiro Matsunaga, Michiko Kimoto, Vanessa Weixun Lim, Hui Pen Tan, Yu Qian Wong, William Sun, Shawn Vasoo, Yee Sin Leo, Ichiro Hirao

**Affiliations:** Institute of Bioengineering and Bioimaging, 31 Biopolis Way, The Nanos, #07-01, Singapore 138669, Singapore; Institute of Bioengineering and Bioimaging, 31 Biopolis Way, The Nanos, #07-01, Singapore 138669, Singapore; National Centre for Infectious Diseases, 16 Jalan Tan Tock Seng, Singapore 308442, Singapore; Institute of Bioengineering and Bioimaging, 31 Biopolis Way, The Nanos, #07-01, Singapore 138669, Singapore; Institute of Bioengineering and Bioimaging, 31 Biopolis Way, The Nanos, #07-01, Singapore 138669, Singapore; Institute of Bioengineering and Bioimaging, 31 Biopolis Way, The Nanos, #07-01, Singapore 138669, Singapore; Department of Physiology, Yong Loo Lin School of Medicine, National University of Singapore, Singapore 117593, Singapore; National Centre for Infectious Diseases, 16 Jalan Tan Tock Seng, Singapore 308442, Singapore; Department of Infectious Diseases, Tan Tock Seng Hospital, 11 Jalan Tan Tock Seng, Singapore 308433, Singapore; Lee Kong Chian School of Medicine, Nanyang Technological University, 59 Nanyang Dr., Experimental Medicine Building, Singapore 636921, Singapore; National Centre for Infectious Diseases, 16 Jalan Tan Tock Seng, Singapore 308442, Singapore; Department of Infectious Diseases, Tan Tock Seng Hospital, 11 Jalan Tan Tock Seng, Singapore 308433, Singapore; Lee Kong Chian School of Medicine, Nanyang Technological University, 59 Nanyang Dr., Experimental Medicine Building, Singapore 636921, Singapore; Saw Swee Hock School of Public Health, National University of Singapore, 12 Science Drive 2, #10-01, Singapore 117549, Singapore; Institute of Bioengineering and Bioimaging, 31 Biopolis Way, The Nanos, #07-01, Singapore 138669, Singapore

## Abstract

Genetic alphabet expansion of DNA by introducing unnatural bases (UBs), as a fifth letter, dramatically augments the affinities of DNA aptamers that bind to target proteins. To determine whether UB-containing DNA (UB-DNA) aptamers obtained by affinity selection could spontaneously achieve high specificity, we have generated a series of UB-DNA aptamers (*K*_D_: 27−182 pM) targeting each of four dengue non-structural protein 1 (DEN-NS1) serotypes. The specificity of each aptamer is remarkably high, and the aptamers can recognize the subtle variants of DEN-NS1 with at least 96.9% amino acid sequence identity, beyond the capability of serotype identification (69−80% sequence identities). Our UB-DNA aptamers specifically identified two major variants of dengue serotype 1 with 10-amino acid differences in the DEN-NS1 protein (352 aa) in Singaporeans’ clinical samples. These results suggest that the high-affinity UB-DNA aptamers generated by affinity selection also acquire high target specificity. Intriguingly, one of the aptamers contained two different UBs as fifth and sixth letters, which are essential for the tight binding to the target. These two types of unnatural bases with distinct physicochemical properties profoundly expand the potential of DNA aptamers. Detection methods incorporating the UB-DNA aptamers will facilitate precise diagnoses of viral infections and other diseases.

## INTRODUCTION

DNA and RNA aptamers are single-stranded oligonucleotides that bind specifically to target molecules, and thus are expected to serve as antibody alternatives. Aptamers are initially obtained by an evolutionary engineering method called SELEX (Systematic Evolution of Ligands by EXponential enrichment) (1,2), involving repetitive cycles of selection and PCR amplification using libraries with randomized sequences. Once the appropriate aptamer sequences are determined, chemical synthesis provides the optimized and functionalized aptamers on a large GMP scale with high QC. Although many DNA and RNA aptamers have been developed (3–8), their practical applications remain limited due to their insufficient affinities (*K*_D_ values 10^–7^−10^–9^ M) (9,10) and specificities to their targets. Thus, over the past few decades, improved methods for aptamer generation have been reported (7,8,11–15).

**Figure 1.**
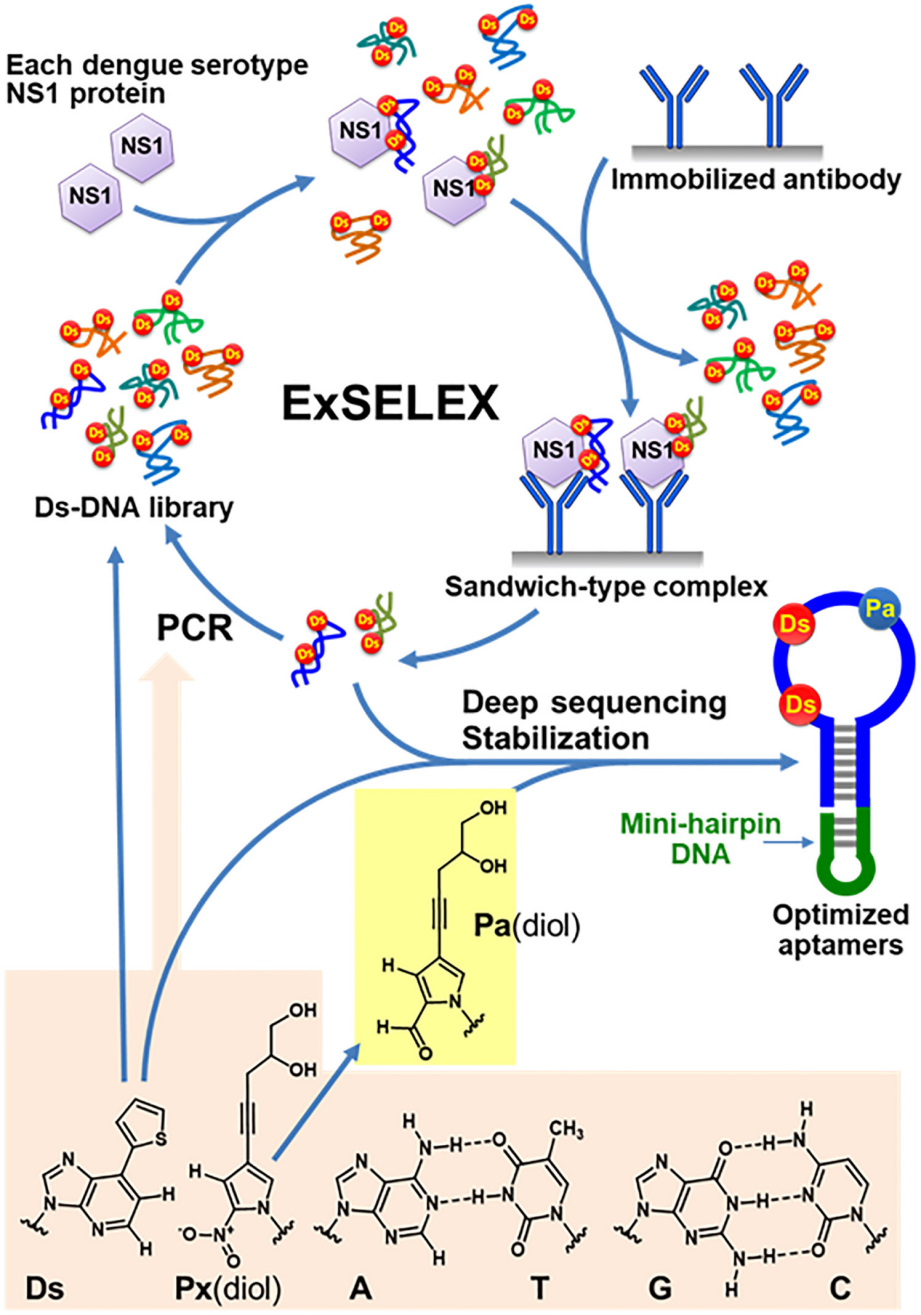
ExSELEX scheme to generate UB-DNA aptamers targeting each DEN-NS1 serotype or sub-serotype variant using an aptamer−antibody sandwich method. The Ds-containing DNA library is mixed with each target DEN-NS1. The DNA–protein complexes are captured with the immobilized anti-NS1 antibody. After washing to remove the unbound DNA species, the bound DNA species are recovered and subjected to PCR amplification involving the Ds–Px pair as a third base pair, to obtain the enriched DNA library for the next round of selection. After several ExSELEX rounds, the sequences in the enriched DNA libraries are determined by deep sequencing, and the aptamer candidates are optimized and stabilized by adding an extraordinarily stable mini-hairpin DNA. One of the aptamer candidates contained the Px base, which resulted from the mutation from the natural base to Px during PCR in ExSELEX. Since the Px nucleoside is unstable under the DNA chemical synthesis conditions, the Px(diol) nucleosides in the aptamers were replaced with the Pa(diol) nucleotide for the synthesis. The Ds–Px pair is a hydrophobic unnatural base pair without any clear hydrogen-bonding interactions between the pairing bases (59).

We recently developed a method to generate high-affinity DNA aptamers containing a hydrophobic unnatural base (UB) (Ds: 7-(2-thienyl)imidazo[4,5-*b*]pyridine) as a fifth letter, by genetic alphabet Expansion for SELEX (ExSELEX) (Figure 1) (16–18). The Ds base specifically pairs with another UB, Px (a diol-modified 2-nitro-4-propynylpyrrole). This Ds−Px pair functions as a third base pair in PCR (19,20), enabling the Ds-containing DNA (Ds-DNA) aptamer generation by ExSELEX. The presence of a few Ds bases in DNA aptamers significantly augments their affinities to the targets (10^–10^−10^–12^ M *K*_D_), by increasing the hydrophobic interactions between the aptamers and target proteins (16,21,22). However, the information for enhancing the specificity of the high-affinity UB-DNA aptamers has remained insufficient.

The relationship between affinity and specificity in aptamer generation has been debated (23–25). In general, aptamers are obtained by iterative affinity selection from randomized sequence libraries. If the aptamer affinity is closely correlated with the target specificity, then the high-affinity aptamers obtained by SELEX spontaneously beget the specificity to their target. Some data support this hypothesis (23), but others do not (24,25). However, the most important issue is the limitation of the aptamer affinities generated by conventional methods using four-letter libraries (9). Only a few reports have described highly specific aptamers, which recognize one or few amino acid differences in their target proteins (26–29). To systematically scrutinize the relationship between affinity and specificity, more information about high-affinity aptamers (less than 10^–9^ M *K*_D_) is required.

To investigate the relationship between aptamer affinity and specificity and to apply UB-DNA aptamers to diagnostics, we present the high-affinity UB-DNA aptamer generation targeting each of the four types (serotypes) of dengue virus non-structural protein 1 (DEN-NS1). Dengue (DENV) is an arthropod-borne flavivirus with four main serological types (DEN1−4). DEN-NS1 proteins are secreted into the blood of infected patients and are one of the most established biomarkers in the early phase (30,31). A secondary infection with a different DENV serotype causes severe diseases, such as Dengue Haemorrhagic Fever and Dengue Shock Syndrome. Thus, the serotype identification of the DENV infected patients may be important in the early diagnostic stage. Among the four serotypes, the sequence identity of DEN-NS1 proteins is 69−80%; however, DNA and RNA aptamer sets that can identify each serotype of DEN-NS1 proteins have not been reported. Some groups have described the generation of antibodies to each NS1 serotype (32–36), but their diagnostic kits are not commercially available. Currently, only an RT-qPCR format is used to identify the DENV serotypes from the viral RNAs extracted from patients’ blood samples. Although the RT-qPCR format has been dramatically advanced as a rapid test in the COVID-19 pandemic, a sandwich method for ELISA has immense potential for a wide range of applications, such as a lateral flow assay (LFA), for simple and rapid Point-of-Care (POC) diagnostic tests.

Here, we present a series of high-affinity UB-DNA aptamers (27−180 pM *K*_D_ values) with remarkably superior specificities to each DEN-NS1 sub-serotype variant beyond the serotype identification. By combining these UB-DNA aptamers with an anti-NS1 monoclonal antibody, we developed a highly specific sandwich-type ELISA (enzyme-linked immunosorbent assay) format for DENV diagnostics, in which the anti-NS1 monoclonal antibody binds to all four serotypes of DEN-NS1. We tested the ELISA format with Singaporean DENV patient sera, and found several mutant NS1 variants within each serotype of these Singaporean patient samples, especially serotypes 1 and 2. Our UB-DNA aptamers recognize each of the variants with at least 96.9% sequence identities at the amino acid sequence level, which are much better than the detection of the differences among the DEN-NS1 serotypes (69−80%). Furthermore, we found that the Px base, which is a pairing partner of Ds, also increases aptamer affinity as a sixth-letter component. One of the UB-DNA aptamers targeting DEN-NS1 serotype 2 (DEN2-NS1) contained Px and Ds bases, which were required for the high-affinity binding. These high-affinity five/six-letter DNA aptamers exhibit robust specificity capable of recognizing a few amino acid mutations, thus opening the door to precise point-of-care diagnostics.

## MATERIALS AND METHODS

### General information for biological experiments and materials

The DNA fragments, including DNA aptamer variants, DNA libraries and primers, were chemically synthesized in-house with an H8 DNA/RNA Synthesizer (K&A Laborgeraete) using phosphoramidites or purchased from Integrated DNA Technologies (IDT). The phosphoramidites of the natural bases were purchased from Glen Research, and the commercially available modified phosphoramidites were purchased from Glen Research, Link Technologies and ChemGenes Corporation. The Ds and diol-Pa phosphoramidites were chemically synthesized in-house, as described previously (37,38). The chemically synthesized DNAs were purified by denaturing gel electrophoresis or directly used without further purification (for some primers and probes from IDT). Unnatural-base substrates (dDsTP, diol-dPxTP, Cy5-dPxTP and dPa′TP) were chemically synthesized, as described previously (20,37,39).

General biological reagents and chemical compounds were purchased from commercial suppliers (Jackson ImmunoResearch, Promega, Thermo Fisher Scientific, Nacalai Tesque, 1^st^ BASE, Promega, Sigma-Aldrich/Merck, New England Biolabs, Bio-Rad Laboratories, KPL, and TCI).

Recombinant DEN-NS1 proteins (polyhistidine-tagged DEN1-NS1, DEN2-NS1, DEN3-NS1 and DEN4-NS1) were purchased from The Native Antigen Company (DEN1-NS1: Nauru/Western Pacific/1974; DEN2-NS1: Thailand/16681/84; DEN3-NS1: Sri Lanka D3/H/IMTSSA-SRI/2000/1266; DEN4-NS1: Dominica/814669/1981). Recombinant Zika virus NS1 proteins (MR 766 Uganda strain and Brazil strain) were obtained from R&D Systems, Inc. and ACROBiosystems. The recombinant DEN1-NS1 variant 2 (with a polyhistidine tag) was prepared in-house, through the ExpiCHO Expression System (Thermo Fisher Scientific) using an expression vector encoding the NS1 region obtained from the PD1-2 patient sample cDNA, followed by purification with Ni-NTA agarose (Thermo Fisher Scientific).

Control human serum (PD0-1) was obtained from a healthy Japanese volunteer who has never been infected by DENV. Whole-blood samples were collected in a Serum Separation Transport Tube or EDTA tubes (Becton Dickinson), from dengue patients referred by the Communicable Disease Centre, TTSH. Blood samples were obtained from patients consenting to the study. Each patient provided a written informed consent. The study protocol was approved by the NHG DSRB (references 2015/00528 and 2016/00076). The recruited patients were tested and confirmed as NS1 positive by routine hospital diagnostics, using an SD BIOLINE Dengue NS1 Ag rapid test (Alere), and had a fever for less than 5 days from illness onset. The DENV infection was confirmed by an RT-qPCR analysis of the samples. Dengue serotypes were also determined by an FTD dengue differentiation RT-qPCR test from Fast Track Diagnostics, using a Bio-Rad CFX96 instrument for the samples, and reconfirmed by Sanger sequencing or IonPGM sequencing of the RT-PCR products. For antigen RNA extraction from patient samples, we used a QIAamp cador Pathogen mini kit (QIAGEN). The serum or plasma samples were obtained during the acute phase (≤7 days post fever) of their dengue infection. The sample information is summarized in Supplementary Table S1.

### Monoclonal antibody production

Monoclonal antibodies Ab#D06 and Ab#D25 were generated by immunization of New Zealand white rabbits with DEN2-NS1 and DEN4-NS1 (The Native Antigen Company). The B cells from the immunized rabbits were FACS-sorted into microtiter plates and screened for binding to NS1 of all four dengue serotypes by ELISA. Antibody genes were recovered from positive B cells by RT-PCR and cloned into expression vectors containing the rabbit IgG framework. Monoclonal antibodies were obtained by transient expression of ExpiCHO cells (Thermo Fisher Scientific) and purified with protein A affinity beads (Pierce).

### ExSELEX

In the first rounds of ExSELEX, we used two or four nmol of the single-stranded DNA libraries (88-mer, 5′-GCACTCCATGATATGGTCTACTG-N_42_-GACAAGCGGAGTAGTTAGACCGT-3′), which are a mixture of 74 sub-libraries. Each sub-library contains two Ds bases at predetermined positions in the 42-nucleotide randomized sequence region. The ExSELEX conditions are summarized in Supplementary Tables S2 and S3. In general, the DNA library, diluted in binding buffer (20 mM Tris–HCl, pH 7.5, 150 mM NaCl, 1 mM MgCl_2_ and 2.7 mM KCl), was denatured by heating at 95°C for 5 min, immediately cooled on ice for 10 min, and then kept at room temperature (25°C) for 10 min. To the diluted DNA library solution, Nonidet *P*-40 (Nacalai Tesque) or Tween 20 was added at the indicated concentrations. The library was incubated with each target protein (DEN1-NS1, DEN2-NS1, DEN3-NS1 or DEN4-NS1 for ExSELEX-1, -2, and -3; DEN1-NS1 variant 2 or clinical serum sample/PD1-13 for ExSELEX-4) (Supplementary Tables S2 and S3) at 25°C in the presence or absence of additives (BSA and human serum, PD0-1). The DNA–NS1 complexes were separated from the unbound DNA species by one of four different methods (Methods A–D). Method A is ultrafiltration with Amicon Ultra Centrifugal Filter Units (MWCO: 100 kDa). Method B is capturing the complexes with an anti-DEN-NS1 antibody, Ab#D06, coated on microtiter plates (MaxiSorp™ 96-well plates from Thermo Fisher Scientific). Method C is a pull-down method, using Dynabeads His-Tag Isolation and Pulldown Magnetic Beads (Thermo Fisher Scientific). Method D is an electrophoresis gel-mobility shift assay. In Methods A, B and C, we washed the captured DNA–NS1 complexes several times at room temperature (∼23°C), and the NS1-bound DNA was recovered by a treatment with 150 mM NaOH, followed by desalting. The recovered DNA was amplified by PCR using forward 5′-PCR and reverse 3′-PCR primers, in the presence of unnatural substrates, dDsTP and diol-dPxTP. The reverse 3′-PCR primer contains a linker and oligo T at the 5′-terminus, to differentiate the lengths of the library and its complementary strands, which allows their separation by denaturing polyacrylamide gel electrophoresis (40). The single-stranded Ds-DNA libraries were isolated and purified by denaturing 8% PAGE, for the next round of selection. From Round 2, to remove the non-specific DNA binding species, pre-counter selections were performed by incubating the DNA library solution with the magnetic beads only or in the antibody-coated wells on the plates, before the target binding. In ExSELEX-1 (Rounds 4–9), ExSELEX-2 (Rounds 4–9), and ExSELEX-4 (Rounds 3–7), to remove the DNA species that bound to the other serotype NS1 proteins, post-counter selections were performed. In these post-counter selections, the DNA solutions, eluted from the DNA–NS1 complexes (before PCR), were incubated with the non-target serotype NS1 proteins at 25°C for 30 min, and then the undesired DNA–NS1 complexes were removed from the solution with magnetic beads. The resultant DNA solutions were subjected to PCR amplification.

### Deep sequencing

The aptamer candidate sequences were determined from the enriched DNA libraries in the final rounds of ExSELEX-1, ExSELEX-2, ExSELEX-3 and ExSELEX-4, by our sequencing method with an Ion PGM system (Thermo Fisher Scientific), as described previously (41). The DNA libraries were amplified by replacement PCR without dDsTP, but with diol-dPxTP or dPa′TP. After the purification of the PCR products, the sequencing samples were prepared by using an Ion Plus Fragment Library Kit with an Ion Express Barcode Adapters 1–16 Kit and an Ion PGM Hi-Q View OT2 Kit, followed by deep sequencing using an Ion PGM Hi-Q View Sequencing Kit (Thermo Fisher Scientific). The obtained sequence data were processed and clustered into families, and the unnatural base positions in the randomized region of each family were estimated by using in-house Perl scripts (41). The sequencing reads and clustered family counts are summarized in Supplementary Table S4.

### Identification of Px in the aptamer strand of D2-1 family

To identify the presence of diol-Px in the D2-1 family clones, we first captured the targeted family sequences from the enriched library in the final round of ExSELEX-3 targeting DEN2-NS1, by using a specific hybridization probe (5′-biotin-CCGCCTCTTGTTCCCAGTCGGAC-3′). The DNA library (100 μl, 50 nM in probing buffer, 20 mM Tris–HCl, pH 7.6, 0.5 M NaCl, 10 mM MgCl_2_) was annealed with the probe (1 μl, 5 μM in water), by heating at 95°C for 3 min, followed by cooling by –0.1°C/s to 60°C, and maintaining the solution at 60°C for 15 min. The mixture was incubated with Hydrophilic Streptavidin Magnetic Beads (New England Biolabs) at 60°C for 5 min. We then collected the magnetic beads, containing the target clones hybridized with the probe, and washed them five times with 150 μl of probing buffer (prewarmed at 60°C). The hybridized clones were recovered from the beads by an incubation with 120 μl of water at 75°C for 5 min. The recovered DNA (100 μl) was subjected to 20-cycle PCR (400 μl) using the forward 5′-PCR primer and the reverse linker-conjugated 3′-PCR primer, in the presence of dDsTP and diol-dPxTP (50 μM each), and the aptamer strand was purified by denaturing PAGE. The binding of the isolated aptamer strand to DEN2-NS1 was confirmed by an electrophoresis gel-mobility shift assay (EMSA). The chemically synthesized D2-1 DNA, D2-1-96(3Ds), in which we added three Ds bases at each position, did not bind to DEN2-NS1, and thus one of the three UB positions might be Px. The isolated aptamer strand (0.5 pmol) was amplified by 8-cycle PCR (25 μl), in the presence of 10 μM Cy5-dPxTP and 50 μM dDsTP with a FAM-labeled 5′-PCR primer and the linker-conjugated 3′-PCR primer, to label the 5′-terminus of the aptamer strand with FAM and the Px-containing strand with Cy5. The PCR products were analyzed by denaturing 15% PAGE, and the band patterns were detected by the FAM and Cy5 fluorescence with a bio-imaging analyzer, ChemiDoc™ MP (Bio-Rad). The PCR using the linker-conjugated 3′-PCR primer revealed that the mobility of the complementary strand of the aptamer sequence became slower than that of the aptamer strand, and we identified both the aptamer and its complementary strands separately on the gel. Both strands of the PCR products from the isolated D2-1 strand emitted Cy5 fluorescence, indicating that the D2-1 aptamer strand contains at least one Px base. The FAM-labeled aptamer strand in the remaining PCR product was purified by denaturing 8% PAGE for further experiments. Since the Px nucleoside is degraded under basic conditions due to the instability of the 2-nitropyrrole nucleoside (42), we utilized this property to identify the Px position based on the results that the DNA fragments partially decompose at the Px position by a concentrated ammonia treatment at 55°C for 4 h. After removing the ammonia solution, the residue was suspended in 20 μl of Hi-Di Formamide (Thermo Fisher Scientific), and 10 μl aliquots were fractionated by denaturing 8% PAGE. The DNA band patterns on the gel were analyzed with a bio-imaging analyzer, LAS4000 (Fuji Film), before and after staining with SYBR Gold. From the digestion pattern on the gel, we assessed the Px position in D2-1. The 5′-FAM fluorescence detection showed one main band corresponding to the 5′-digested fragment (∼57-mer), and the detection by SYBR Gold staining showed two bands corresponding to the 5′- (∼57-mer) and 3′-digested (∼39-mer) fragments. These digestion patterns indicated that the DNA fragment contains one Px base at the third unnatural base position (position 57) from the 5′ end (Supplementary Table S5).

### Preparation of the authentic D2-1, D2-1y-96

The authentic D2-1 aptamer, D2-1y-96, was prepared by using two chemically synthesized fragments (5-half: 5′-ACTCCATGATATGGTCTACTGGTCCG-Ds-CTGGGAACAAG-Ds-GGCGGGAGGGA-3′, 3-half: 5′-GGTCTAACTACTCCGCTTGTCGCACCCACACCC-Ds-TCCCTCCCGCC-3′, the complementary sequences are underlined), via primer extension and PCR amplification. The primer extension (100 μl) was performed by using 2 μM of each of the 5-half and 3-half fragments in the presence of 50 μM diol-dPxTP, followed by purification using a QIAquick Gel Extraction Kit (QIAGEN). Using the primer-extended product as the template, 8-cycle PCR was performed in the presence of 50 μM concentrations of both dDsTP and diol-dPxTP, and the aptamer strand was purified by denaturing 8% PAGE. The binding of the prepared aptamer strand, D2-1y-96, was analyzed by SPR and EMSA.

### Electrophoresis gel-mobility shift assays (EMSA)

For the DNA folding, the DNA fragments diluted in binding buffer were heated at 95°C for 5 min, followed by immediate cooling on ice for 10 min. The DNA solution (50 nM) was mixed with or without the respective NS1 protein (25 nM as the hexamer form; total 150 nM monomeric units) in binding buffer supplemented with 0.05% Nonidet *P*-40, and incubated at 25°C for 30 min. After the incubation, the samples were mixed with glycerol (final concentration 5%), and the complex formation was analyzed by PAGE (4% polyacrylamide gel containing 44.5 mM Tris-borate, 1 mM MgCl_2_, 2.7 mM KCl, 5% glycerol, with or without 2 M urea). Gel electrophoresis was performed at 26–28°C for 50 min, in the constant temperature mode (at a 3 W setting with a temperature probe set at 30°C). The DNA band patterns on the gels were detected with the LAS-4000 imager, after staining with SYBR Gold. The band densities corresponding to free DNAs were quantified using the Multi Gauge software, to determine the relative shifted ratios for comparing the degrees of complex formation.

### Surface plasmon resonance (SPR) analysis

Binding affinity profiles were obtained at 25°C on a Biacore T200 (GE Healthcare), using running buffer (binding buffer supplemented with 0.05% Tween 20). For the immobilization of each ligand (aptamer variant), we used streptavidin-coated sensor chips and immobilized the biotinylated aptamer variant on the flow cell, by injecting 0.5 nM of the ligand solution in running buffer at a flow rate of 0.5 μl/min for 960 sec. For some of the Ds-DNA aptamers (D1 and D2 aptamer variants), we found that the immobilization in the presence of NS1 gave reproducible target binding profiles, which would result from the aptamer immobilization at an appropriately separated distance, to ensure efficient binding with the multimeric NS1 proteins. Binding kinetic profiles were monitored by injecting at least five different concentrations of the analyte solutions (0.625–20 nM) for 150 s (binding), at a flow rate of 30 μl/min. The analyte dissociation patterns were then recorded for 600 or 1200 s (for D1-1-48h, D2-1d-72 h, D3-2-59h, and D4-3-57h). To regenerate the ligand on the flow cell surface, a denaturation solution (50 mM NaOH) was injected for 5 s, and then the ligand was equilibrated in running buffer for 10 min. The kinetic parameters for the target binding, association rates (*k*_on_), dissociation rates (*k*_off_), and dissociation constants (*K*_D_ = *k*_off_/*k*_on_) were determined with the BIAevaluation software, version 3.0, by using the double-reference subtraction method and global curve fitting (more than twice at each concentration) to a 1:1 Langmuir model.

### ELISA using aptamer−antibody pair (Apt/Ab ELISA)

All incubations were performed at room temperature. Microtiter plates were coated overnight with 100 μl/well of 10 μg/ml streptavidin in 0.1 M sodium carbonate buffer (pH 9.6). The streptavidin-coated wells were blocked with 300 μl of 10 mg/ml BSA in 1× D-PBS for 2 h, and then the wells were washed three times with 200 μl of washing buffer (20 mM Tris–HCl, pH 7.5, 150 mM NaCl, 1 mM MgCl_2_, 2.7 mM KCl, 0.05% Tween 20). Each UB-DNA aptamer was immobilized on the streptavidin-coated wells by a 2-h incubation with 100 μl of 5−15 nM UB-DNA aptamer [D1-1-48 h (AptD1), 19D1F1-3 (AptD1b), D2-1d-72h (AptD2), D3-2-59h (AptD3) or D4-3-57h (AptD4)] in dilution buffer (washing buffer supplemented with 1 mg/ml BSA), and then each well was washed three times with 200 μl of washing buffer. To the aptamer-coated wells, 100 μl of an NS1–Ab#D06 mixture solution was added and incubated for 30 min. The solutions were prepared beforehand at 1:9 ratios (vol/vol), by a 30-min incubation of each NS1 protein in dilution buffer or human serum (PD0-1)/plasma with 11.1 nM of Ab#D06 in dilution buffer, supplemented with Tween 20 at a 2% final concentration (dilution buffer 2). After washing the wells once, 100 μl of secondary detector solution (anti-rabbit IgG HRP conjugate, diluted 1: 2,500 with dilution buffer) was added to each well, and then incubated for 30 min. The wells were washed six times, and then 100 μl/well of TMB-substrate solution was added and the plates were incubated for 30 min. After adding 100 μl of 1 N HCl to the wells to stop the reaction, the absorbance at 450 nm (OD_450_) was measured with a microplate reader, Cytation 3 (BioTek). The assays under each condition were performed in duplicate (*n* = 2), and the average absorbance data are shown in the graphs with error bars, which represent one average deviation. When the signal in at least one of the two sample wells was saturated (OD_450_ > 4.000), the data are shown with wavy lines in the graphs.

### ELISA using an antibody−antibody pair (Ab/Ab ELISA)

The Ab/Ab ELISA was performed in a similar manner to the Apt/Ab ELISA, with some modifications. Instead of the aptamer-coated plates, the antibody-coated plates were prepared by a 2-h incubation with 2 μg/ml Ab#D25 (100 μl/well) in 0.1 M sodium carbonate buffer (pH 9.6), followed by blocking with BSA. To prepare the NS1–Ab#D06 mixture solutions with dilution buffer 2, biotinylated Ab#D06 was used. For biotinylation, the Ab#D06 solution (6.67 μM in 1× D-PBS) was mixed with Thermo Scientific™ EZ-Link™ Sulfo-NHS-LC-Biotin (final concentration 117 μM), and the mixture was incubated at room temperature for 30 min. The antibody was then recovered after desalting, using Amicon Ultra-0.5 Centrifugal Filter Units (MWCO: 50 kDa). The biotinylated Ab#D06 solution in 1× D-PBS was kept at 4°C until use. The secondary detector was a streptavidin-HRP conjugate, diluted 1:20 000 with dilution buffer, instead of the anti-rabbit IgG HRP conjugate.

### DNA sequencing of the dengue NS1 region of the extracted RNA samples

To determine the amino acid sequences of NS1 in the clinical samples, we performed sequencing analyses of the DENV NS1 gene RT-PCR products, using Sanger capillary sequencing (for PD1-2, PD1-3, PD2-1, PD2-2, PD2-3, PD3-1, PD3-2, PD3-3, PD3-4 and PD4-1) or multiplex PCR followed by deep sequencing (for the other patient samples), with some modifications of the published protocol (43). RNA from the clinical samples was reverse transcribed into cDNA using Superscript III RNase H(–) Reverse Transcriptase (Thermo Fisher Scientific) and specific primers or random hexamers. The resulting cDNA was then used as the template for PCR amplification with either *Taq* DNA polymerase (New England Biolabs), AccuPrime *Pfx* DNA polymerase (Thermo Fisher Scientific) or Q5 High-Fidelity DNA polymerase (New England Biolabs). The PCR products were purified from the agarose gels by using a QIAquick gel extraction kit (QIAGEN) or a general silica-gel column type PCR purification kit. The purified products were subjected to a cycle sequencing reaction with a BigDye™ Terminator v3.1 Cycle Sequencing Kit (Thermo Fisher Scientific) or deep sequencing with an Ion PGM system (Thermo Fisher Scientific), following the manufacturer's instructions. The capillary sequencing was performed on a 3500 Genetic Analyzer (Thermo Fisher Scientific), and the sequence reads were assembled manually. The sequencing reads obtained with the Ion PGM system were mapped and analyzed using reported NS1 sequences as references, with the CLC Genomics Workbench software (CLC bio).

### G-to-A scanning

To investigate G-quartet formation and the importance of the G-tract regions in AptD2, we chemically synthesized AptD2 without the mini-hairpin sequence, AptD2-1 (63-mer, 5′-GGCTGGTCCGxCTGGGAACAAGxGGCGGGAGGGAdGGGTGTGGGTGCGACAAGCGGACCAGCC-3′, x = Ds, d = diol-Pa), and its G-to-A variants (AptD2-1a: G15A; AptD2-1b: G25A; AptD2-1c: G28A; AptD2-1d: G32A; AptD2-1e: G37A; AptD2-1f: G43A; and AptD2-1g: G48A), followed by purification using denaturing PAGE. Binding of the purified DNA fragments to DEN2-NS1 was analysed by EMSA, besides UV spectroscopy (220 nm to 400 nm) and thermal-melting profiling (260 nm and 295 nm). We recorded UV spectra and UV melting profiles of the aptamer variants using a SHIMADZU UV-2600 spectrometer equipped with a temperature controller. In the UV spectra analysis, the absorbance of each sample (2 μM in binding buffer) was monitored at 15°C, ranging from 220 nm to 400 nm, and normalized by the maximum absorbance (as 1) and the baseline (as 0). To compare the spectra differences among the original aptamer and G-to-A aptamer variants, each normalized spectrum was divided by that of the original aptamer, AptD2-1, and the ratios were plotted against the wavelength. In the thermal-melting profiling, the absorbances at 260 and 295 nm were monitored at 260 and 295 nm from 15°C to 90°C, at a heating rate of 0.5°C/min. To compare the melting profile differences among the original aptamer and its variants, we normalized the absorbance at 15°C (as 1), and the absorbance changes were plotted against the temperature.

## RESULTS

### UB-DNA aptamer generation targeting each DEN-NS1 serotype

To generate Ds-DNA aptamers targeting each DEN-NS1 serotype, we performed the ExSELEX procedure multiple times (Supplementary Table S2) using a Ds-predetermined sub-library system (16,21,22,44) composed of a mixture of 74 sub-libraries. Each sub-library contained two Ds bases at different defined positions in the 42-natural-base randomized sequence region (16,22). As the targets, four serotypes of DEN-NS1 proteins fused with a His-tag were purchased from The Native Antigen Company (Oxford, UK), and the amino acid sequence identities among the DEN-NS1 serotypes are 69−80% (Supplementary Figure S1).

In addition to the conventional SELEX procedure using the complex size separation by ultrafiltration and EMSA or the His-tag capture method, we employed a selection method using an anti-DEN-NS1 monoclonal antibody (Ab#D06), which binds to all four serotypes of DEN-NS1 with 27–107 pM *K*_D_ values: 107 pM for serotype 1 (DEN1-NS1), 65 pM for serotype 2 (DEN2-NS1), 75 pM for serotype 3 (DEN3-NS1), and 27 pM for serotype 4 (DEN4-NS1) (Supplementary Figure S2). The Ds-DNA library was mixed with each DEN-NS1 serotype, and then the NS1−DNA complexes were captured with the Ab#D06 antibody immobilized on a plate (Figure 1). In this antibody-capture method, the aptamers are isolated as the ternary complexes of Ab#D06−NS1−aptamer, and thus they can be directly used in a sandwich-type ELISA format. The unbound DNA species were washed from the plate, and the DNA species bound to the NS1−antibody complexes on the plate were isolated and amplified by PCR.

For subsequent rounds of selection, the Ds-strand libraries were separated from the PCR-amplified duplex DNA libraries according to our method using the reverse 3′-PCR primer conjugated to an additional oligo T via a triethylene glycol (TEG) linker (16,40). The additional oligo T region of the primer is not replicated due to the linker (45), resulting in the longer length of the complementary strand than that of the Ds-strand, which can be separated on the denaturing gel. Furthermore, since the reverse 3′-PCR primer contains two consecutive biotinylated thymidines at the 5′-termius of the oligo T, the biotinylated complementary strands are also removed by streptavidin treatment.

The binding of the library to each target in the selection step was performed at 25°C for 5, 15, 30 or 60 min in buffer (20 mM Tris–HCl, pH7.5, 150 mM NaCl, 1 mM MgCl_2_, 2.7 mM KCl in the presence of 0.005% Nonidet *P*-40, 0.05% Tween 20 or 2% Tween20). The selection and washing conditions and PCR cycles in each round were determined based on the quantitative PCR (qPCR) analysis (Supplementary Figure S3), prior to the large-scale PCR amplification of the isolated libraries. In each round, the qPCR was performed using a small portion of the isolated library solution obtained by the selection step, with or without the target proteins. From the qPCR amplification profile, we determined the appropriate cycle number for the large-scale PCR. By comparing the qPCR profiles with those of the selection without the target, we determined the selection and washing conditions for the following round and gradually increased the selection pressure. In addition, we performed each round of selection in the presence of gradually increased concentrations of human serum to adapt the conditions for ELISA tests using clinical serum samples.

After 7−10 rounds of selection (Supplementary Table S2), we obtained enriched DNA libraries and confirmed their high specificities to each DEN-NS1 serotype by electrophoresis gel-mobility shift assays (EMSAs) (Supplementary Figure S4). The high Ds dependency was confirmed by EMSAs using each Ds→natural base variant of the enriched libraries, prepared by our PCR-mutagenesis (replacement PCR) method (16,41). These Ds→natural base variants did not form clearly discernible complexes with any DEN-NS1 proteins (Supplementary Figure S4). The sequences in the enriched DNA libraries were determined by our UB-sequencing methods using the Ion PGM system (Supplementary Table S4 and Supplementary Figures S5−S8) (21,41), and from the sequence families, we selected several aptamer candidates for each DEN-NS1 serotype (Supplementary Table S5).

### Six-letter UB-DNA aptamers targeting DEN2-NS1

A notable case involved the discovery of six-letter UB-DNA aptamers within some candidates obtained by ExSELEX targeting serotype 2 of the DEN-NS1 protein (DEN2-NS1). One of the sequence families (D2-1), which exhibited the highest affinity to DEN2-NS1, contained two Ds and one Px bases (Supplementary Figure S6 and Table S5), and all three bases were required for the high affinity. The DNA fragments (D2-1 family), which were isolated from the enriched library using a specific probe and amplified by PCR in the presence of dDsTP and dPxTP (21), efficiently bound to DEN2-NS1 (D2-1y-96 in Supplementary Table S5). This additional Px in the D2-1 family most likely resulted from the mutation of the natural bases to Px in the aptamer strands during the PCR amplification in ExSELEX.

Initially, we chemically synthesized several aptamer candidates containing two or three Ds bases in the three UB-suspected positions, according to the D2-1 sequence data obtained by our UB-DNA sequencing method (41). However, the EMSA experiments did not detect any binding of these Ds-DNA candidates to DEN2-NS1 (D2-1-96(3Ds) in Supplementary Figure S9B and Supplementary Table S5). Therefore, to identify the UBs in D2-1, we amplified the isolated D2-1 clone, as well as the initial Ds-DNA library as a control, by PCR in the presence of dDsTP and Cy5-conjugated dPxTP (Cy5-dPxTP), using the 5′-primer labelled with FAM and the 3′-primer conjugated with oligo T via the TEG linker (Supplementary Figure 9A), and analysed each of the amplified aptamer and complementary strands on a denatured gel (Supplementary Figure S9C). Although no Cy5 fluorescence was observed in the aptamer strand of the initial DNA library, the aptamer strand of the isolated D2-1 clone was detected with Cy5 fluorescence, suggesting the Px incorporation into the aptamer strand. Thus, we developed the method to determine the Px position in DNA (Supplementary Figure S9D).

We determined the Px position in the D2-1 clone by applying the instability of the Px nucleoside under basic conditions. By using the instability of the Px nucleoside, the 5′-FAM-labelled clone, amplified by PCR using the FAM-labelled 5′-PCR primer, was partially digested at Px positions by the treatment with concentrated ammonia at 55°C. The digested fragments were analysed by denaturing gel electrophoresis (Supplementary Figure S9D). As the results, the digestion of the DNA fragments at position 57, among the three possible UB positions, was clearly observed by FAM detection for the 5′-FAM strand and SYBR detection for both the 5′- and 3′-strands. Thus, we concluded that D2-1 contains two Ds and one Px bases at positions 33, 45 and 57, respectively. This sequence assignment was confirmed by the chemical synthesis of D2-1.

The Px-containing DNA fragments cannot be chemically synthesized, because the Px nucleoside is unstable under the basic conditions employed in the conventional chemical DNA synthesis. This instability results from the combination of the nitro group and pyrrole ring of Px (42), and thus we replaced the nitro group with an aldehyde group (Pa, pyrrole-2-carbaldehyde) (37) for DNA chemical synthesis (Figure 1 and Supplementary Table S5). We synthesized the phosphoramidite derivative of the diol-conjugated Pa nucleoside for DNA chemical synthesis (38), and prepared several D2-1 variants containing Ds and Pa (Supplementary Table S5) by the standard phosphoramidite method.

**Figure 2.**
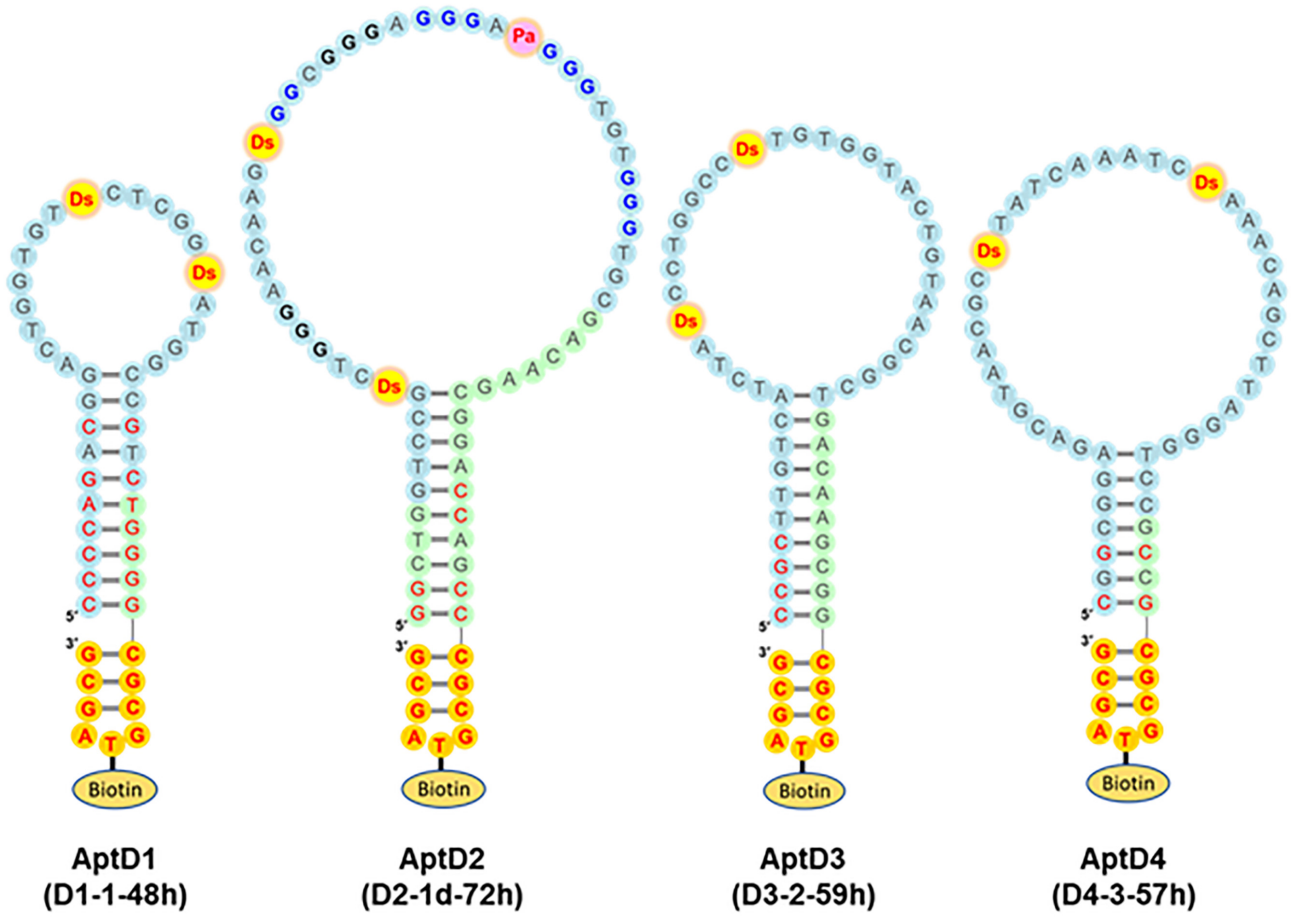
Presumed secondary structures of the optimized UB-DNA aptamers that bind specifically to each DEN-NS1 serotype. AptD1 (D1-1-48h) binds to DEN1-NS1 (variant 1), AptD2 (D2-1d-72h) to DEN2-NS1 (variant 1), AptD3 (D3-2-59h) to DEN3-NS1, and AptD4 (D4-3-57h) to DEN4-NS1. All aptamers contain two Ds bases, while AptD2 contains one Pa and two Ds bases, which are essential for tight binding to the target. The Ds and Pa bases are shown in red with yellow and pink circles, respectively. AptD2 has several G-tract regions, shown in bold, and the four G-tract regions in blue are involved in the G-quadruplex formation. The sequences in the random region and the constant primer regions are shown with light-blue and light-green circles, respectively, and the sequences intentionally mutated to stabilize base pairing are shown in red. The mini-hairpin DNA sequences, CGCGTAGCG, are shown in red with orange circles. The thymidines within the mini-hairpin DNA sequences are used as the biotinylation sites.

The D2-1 family containing one Pa/Px and two Ds bases exhibited higher affinity to DEN2-NS1 (*K*_D_ = 41−114 pM) (Supplementary Table S5) than those of the other families (D2-2 to D2-6) with only two Ds bases. The Px→Pa aptamer variants of D2-1 still retained the high affinity and specificity to DEN2-NS1, suggesting the importance of the diol group of Px for the binding. Accordingly, we developed AptD2 (D2-1d-72h), as an optimized DEN2-NS1 binder (Figure 2).

### Aptamer optimization and ELISA format

We finalized each aptamer sequence: AptD1 (D1-1-48h) for DEN1-NS1, AptD2 (D2-1d-72h) for DEN2-NS1, AptD3 (D3-2-59h) for DEN3-NS1, and AptD4 (D4-3-57h) for DEN4-NS1 (Figure 2), through EMSA screening using their chemically synthesized aptamers and their variants (Supplementary Table S5). Most of the sequences in each family contained a consensus sequence flanked by complementary motifs that form stem-loop structures (Supplementary Figures S5–S8). The binding experiments using deletion variants of each candidate revealed that these stem and loop structures are essential for the tight binding to each target (Supplementary Table S5). Thus, we trimmed and optimized these complementary motifs to form a clear stem structure. Interestingly, when we successfully generated high-affinity aptamer, the stem regions in these aptamers were always observed at their termini (16,17,21,22). In addition, to increase the thermal and enzymatic stabilities and facilitate the immobilization of the aptamer candidates, we added a thermally stable biotin-conjugated DNA sequence (mini-hairpin DNA) (46–48), CGCG(Biotin-T)AGCG, at their 3′-termini (44,49,50). We chemically synthesized these optimized UB-DNA aptamers for further experiments.

**Figure 3.**
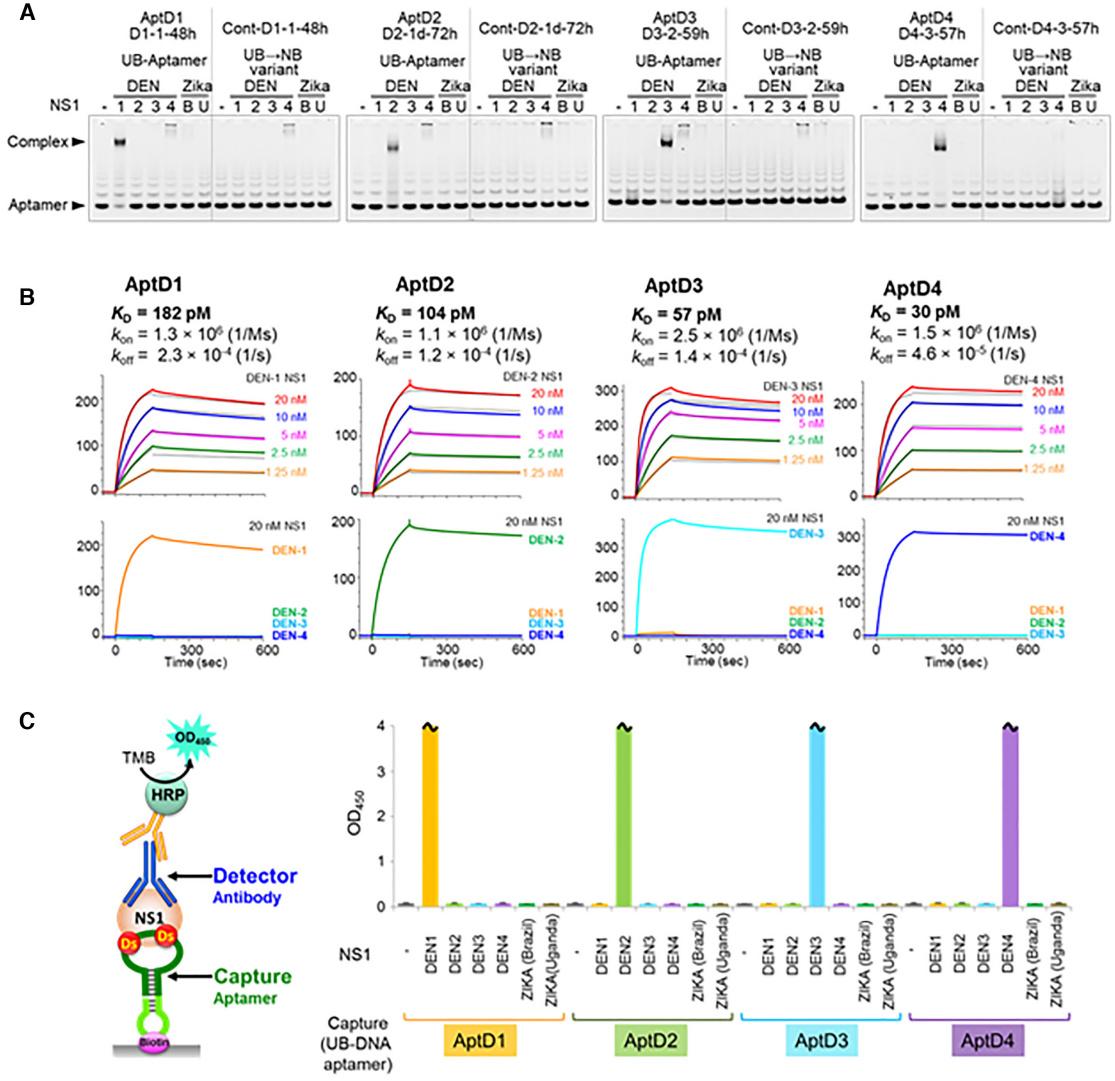
UB-DNA aptamers, D1-1-48h (AptD1), D2-1d-72h (AptD2), D3-2-59h (AptD3), and D4-3-57h (AptD4), specifically recognize the targeted DEN-NS1, allowing for serotype-specific dengue diagnostics. (**A**) Electrophoresis gel-mobility shift assay (EMSA) of the aptamer−NS1 complex formation using each UB-DNA aptamer and their variants without unnatural bases. (**B**) Binding analysis of the UB-DNA aptamers to each DEN-NS1 by a Biacore T200 SPR system at 25°C. Each aptamer's kinetic binding parameters, dissociation constant (*K*_D_), and association and dissociation rates (*k*_on_ and *k*_off_) to each targeted DEN-NS1 were determined. (**C**) Serotype-specific DEN-NS1 detection by ELISA in combination with the UB-DNA aptamer and antibody (Ab#D06) pair. In ELISA, a 10-μl portion of a 100 ng/ml solution of each flavivirus NS1 protein (DENV serotypes 1–4 NS1 proteins, and Zika virus NS1 proteins of a Brazilian strain and a Ugandan strain, purchased from The Native Antigen Company) was used in buffer. The sample size is two per each combination set, and the error bars represent one average deviation. The bars with wavy lines indicate that the signal in at least one of the two sample wells was saturated (OD_450_ > 4.000).

The high specificity of each aptamer to its serotype-specific DEN-NS1 was confirmed by EMSA (Figure 3A) and surface plasmon resonance (SPR) analyses (Figure 3B). The UB→natural-base variants, in which the UBs were replaced with natural bases, showed significantly reduced affinities to each target, indicating the necessity of these UBs for the aptamers’ binding capabilities (Figure 3A). The *K*_D_ values of AptD1, AptD2, AptD3 and AptD4 to their target DEN-NS1 were 182, 104, 57 and 30 pM, respectively (Figure 3B).

The detection of each DEN-NS1 serotype was examined by a sandwich-type ELISA format, using the antibody Ab#D06 as the primary detector agent and the aptamers as capture agents (Figure 3C). The signal was detected by the colorimetric output, using a secondary anti-IgG HRP-conjugated antibody. The ELISA format using the aptamer−antibody sandwich system was optimized according to our reported method, including the determination of the ideal amounts of immobilized aptamers (10). Each aptamer specifically detected its target DEN-NS1 serotype, and no cross-reactivities with non-target DEN-NS1 serotypes or two strains of Zika NS1 proteins were observed (Figure 3C). The limit of detection (LOD) values in buffer and in 10% human serum (control, PD0-1) were 1.19–2.36 ng/ml and 1.14–3.31 ng/ml, respectively, for each DEN-NS1 (Supplementary Figure S10), reaching the sufficient sensitivity required for practical use of this ELISA method. The concentrations of DEN-NS1 secreted in patient blood are the range of low ng/mL to several hundred ng/mL (30,51).

### G-quartet structure of aptamer AptD2

**Figure 4.**
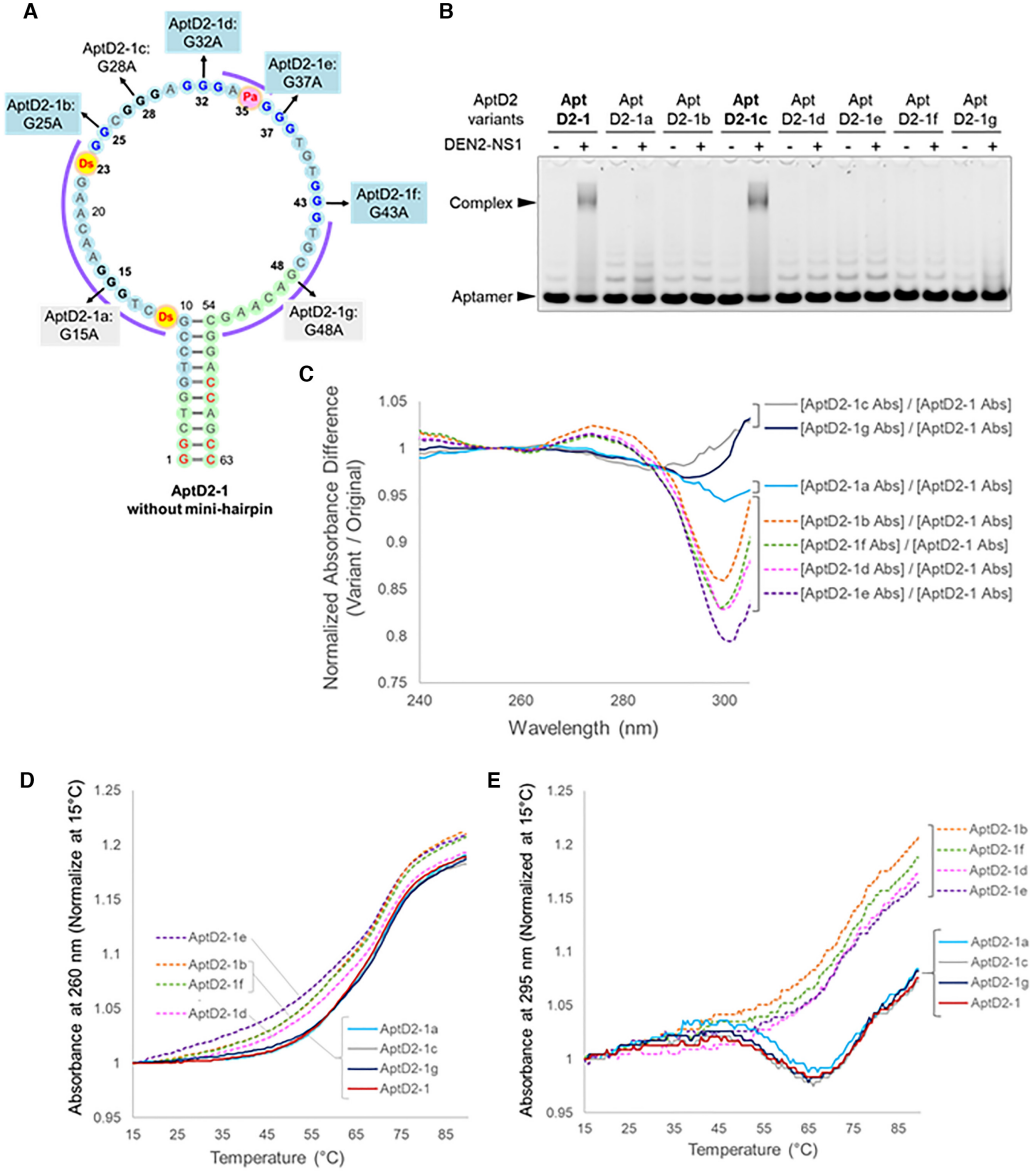
Identification of G-tract regions in AptD2 involved in G-quadruplex formation by the G-to-A scanning method. (**A**) Presumed secondary structure of the 63-mer AptD2-1. In the series of aptamer variants, AptD2-1a through AptD2-1g, a G base in each G-tract region is replaced with A. (**B**) EMSA of the aptamer complex formation with DEN2-NS1 (50 nM as the hexamer form) using each AptD2-1 variant (50 nM). (**C**) UV spectra difference of each AptD2-1 variant at 15°C. (**D**) UV melting profiles at 260 nm of AptD2-1 and its variants. (**E**) UV melting profiles at 295 nm of AptD2-1 and its variants.

AptD2, targeting DEN2-NS1, contained six G-tracts in the loop region (Figure 2), suggesting the formation of a G-quadruplex structure. Therefore, we examined the possibility of a G-quadruplex and identified which G-tracts in AptD2 are involved in the G-quartet. To this end, we developed a G-to-A scanning method using a series of one-point G→A variants of AptD2-1 (63-mer): AptD2-1a to AptD2-1g for the G15A, G25A, G28A, G32A, G37A, G43A or G48A single mutation (Figure 4A). This is because the absorbance at around 300 nm of the Ds base overlaps the G-quartet-specific absorbance at 295 nm, making it difficult to use the conventional spectral analysis for G-quartet structures (52). Thus, we compared the binding abilities to DEN2-NS1, UV spectra, and thermal melting profiles of these G→A variants with those of AptD2-1.

Using this G-to-A scanning, we identified the G-quadruplex composed of the four G-tracts (G24-25, G31-33, G36-38 and G42-44). The EMSA test revealed that only AptD2-1c (G28A) bound to DEN2-NS1, indicating that the other six Gs (G15A, G25A, G32A, G37A, G43A and G48A) are essential (Figure 4B). The relative absorbances of four variants, AptD2-1b, -1d, -1e and -1f, which were normalised with the absorbance of AptD2-1, exhibited significantly different patterns at around 300 nm, as compared to those of AptD2-1a, -c and -g (Figure 4C). These four variants also caused the broadening of their thermal melting profiles monitored at 260 nm, as compared to those of AptD2-1 and the other three variants (AptD2-1a, -1c, and -1g), suggesting the thermal destabilization of the structure (Figure 4D). Finally, the melting profiles at 295 nm of AptD2-1 and three variants (AptD2-1a, -1c, and -1g) provided the characteristic patterns specific to a G-quartet (52) (Figure 4E). These results support the formation of at least two G-quartets among the G24-25, G31-33, G36-38, and G42-44 regions in AptD2-1.

### Serotype-specific detection of DEN-NS1 in patient samples

**Figure 5.**
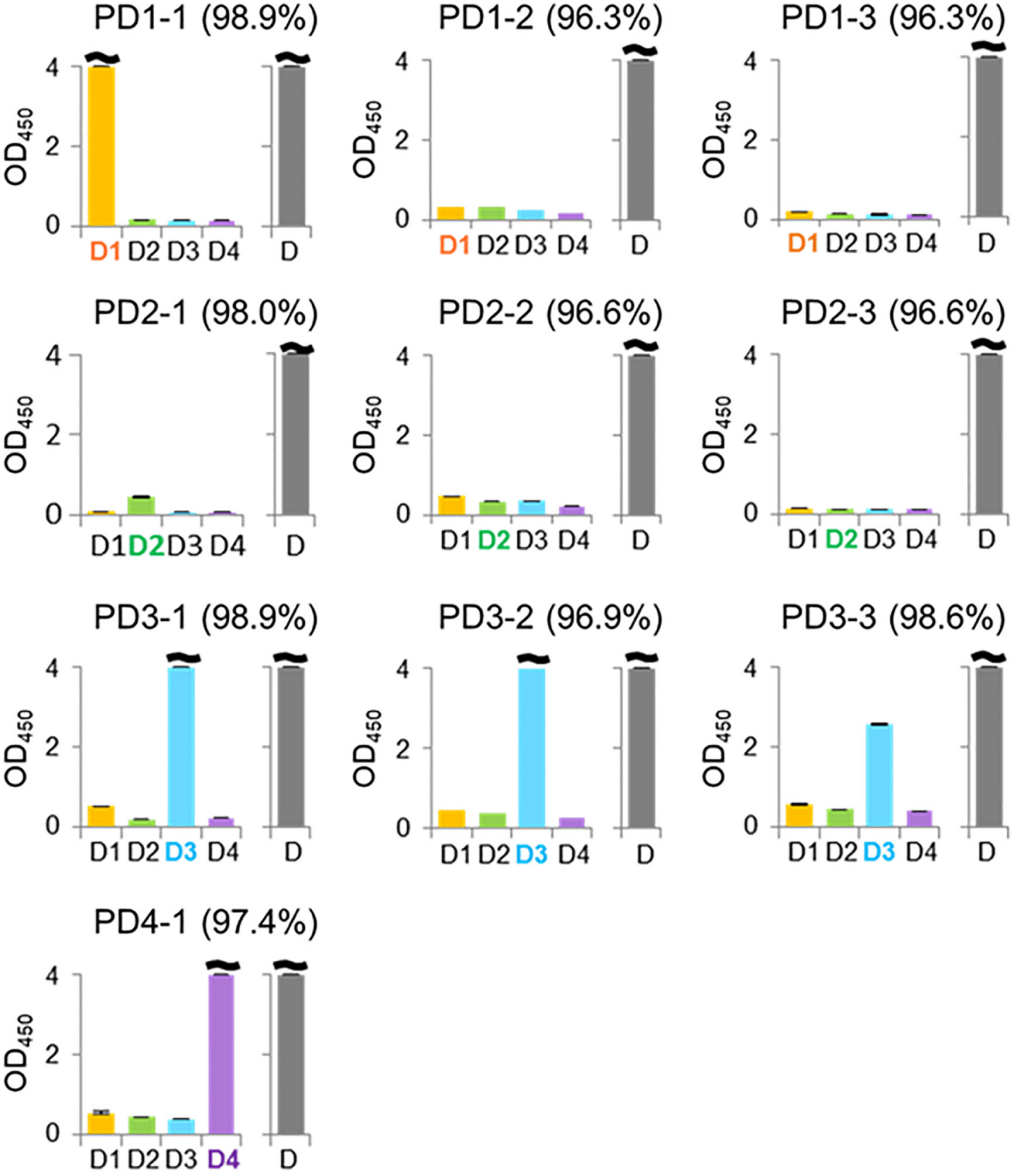
DEN-NS1 detection in ten Singaporean clinical samples by the ELISA formats with the aptamer−antibody sandwich pair and the antibody−antibody pair. The DEN-NS1 detection was performed by the ELISA formats, using aptamer−antibody (Ab#D06) pairs (indicated as D1−D4) and an antibody (Ab#D25)−antibody (Ab#D06) pair (indicated as D). The values (%) in parentheses are the amino-acid sequence identities with each DEN-NS1 serotype purchased from The Native Antigen Company (Supplementary Figure S11).

Using blood samples from ten Singaporean DENV patients (PD1-1 to PD4-1) obtained within 3−5 days after fever onset (patient samples indicated with asterisks in Supplementary Table S1), we evaluated the sensitivity and specificity of our ELISA format for detecting each DEN-NS1 serotype (Figure 5). The dengue serotype of each sample was initially identified by an FTD dengue differentiation RT-qPCR test from Fast Track Diagnostics. We also determined the DEN-NS1 sequences in each patient sample by RT-PCR and sequencing of the mRNA encoding DEN-NS1 (Supplementary Figure S11). Using these conventional sequencing methods, we identified PD1-1, PD1-2 and PD1-3 as serotype 1 (DEN1-NS1), PD2-1, PD2-2 and PD2-3 as serotype 2 (DEN2-NS1), PD3-1, PD3-2 and PD3-3 as serotype 3 (DEN3-NS1), and PD4-1 as serotype 4 (DEN4-NS1). The sequence analysis of these clinical samples revealed several amino acid mutations of DEN-NS1 in each serotype, and some were different from those of the ExSELEX-target DEN-NS1 purchased from The Native Antigen Company.

Our ELISA format detected each DEN-NS1 serotype in the PD1-1, PD2-1, PD3-1, PD3-2, PD3-3 and PD4-1 serum samples (Figure 5). However, PD1-2, PD1-3, PD2-2 and PD2-3 could not be detected. The ELISA format using the antibody−antibody sandwich pair of Ab#D06 and Ab#D25 (1.6–138 pM *K*_D_ values for DEN-NS1, Supplementary Figure S2), and the commercial LFA system (SD BIOLINE) detected DEN-NS1 in all of the samples (Figure 5 and Supplementary Table S1), although these tests could not identify their serotypes.

**Figure 6.**
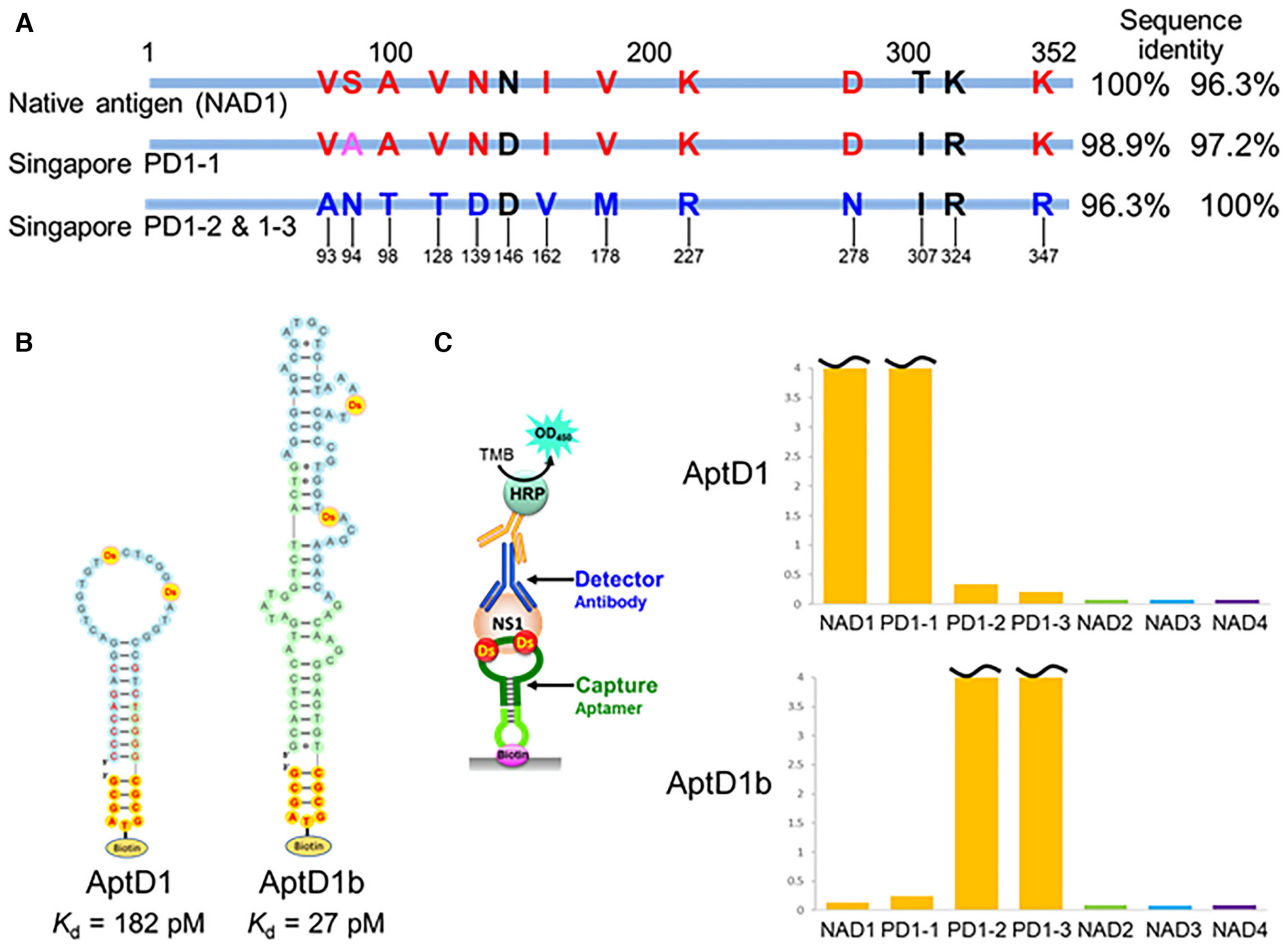
UB-DNA aptamers recognize each DEN1-NS1 variant. (**A**) Differences in the amino acid sequences of DEN1-NS1 proteins derived from the clinical samples (PD1-1, PD1-2 and PD1-3) and the original DEN1-NS1 target from The Native Antigen Company (NAD1). The different amino acids between DEN1-NS1 variant 2 (from PD1-2/PD1-3) and DEN1-NS1 variant 2 from PD1-1 are indicated in red. The sequence identities are presented as compared to NAD1 (left column) and PD1-2/1-3 (right column). (**B**) Presumed secondary structures of UB-DNA aptamers, AptD1 and AptD1b, which specifically target the original DEN1-NS1 variant 1 (NAD1) and a recombinant DEN1-NS1 variant 2 protein (from PD1-2/PD1-3), respectively. The kinetic binding parameters, dissociation constant (*K*_D_), of each aptamer to each cognate DEN1-NS1 variant were determined by an SPR analysis (Supplementary Figure S14). (**C**) DEN-NS1 detection by the aptamer−antibody (Ab#D06) ELISA formats using AptD1 (D1-1-48h) and AptD1b (19D1F1-3). As the test samples (10 μl), different clinical sera (PD1-1, PD1-2 and PD1-3) and each serotype DEN-NS1 recombinant protein from The Native Antigen Company (NAD1, NAD2, NAD3, and NAD4; 100 ng/ml) were used.

These differences in the detection sensitivities among the samples are strongly correlated with the subtle amino-acid differences in each DEN-NS1 serotype. Among the patient samples, only DEN-NS1 proteins with higher amino-acid sequence identity (>96.9%) to the target DEN-NS1 (NAD1), purchased from The Native Antigen Company, were detected by each of our aptamers (Figure 5 and Supplementary Figure S11). The DEN1-NS1 of PD1-1 shared 98.9% sequence identity with NAD1, as detected by ELISA using AptD1. In contrast, PD1-2 and PD1-3 had 96.3% sequence identities with NAD1 and were not detected with AptD1. Similarly, the sequence identities of the DEN2-NS1 of PD2-1, PD2-2 and PD2-3 with that of The Native Antigen Company (NAD2) were 98.0, 96.6 and 96.6%, respectively, and AptD2 barely detected only the PD2-1 sample with the highest sequence identity among these three variants. For DEN3-NS1 and DEN4-NS1, the sequence identities with each NAD were 96.9−98.9%, and AptD3 and AptD4 detected each DEN-NS1. These results suggested that our UB-DNA aptamers potentially have high specificity to targets. There are 10 amino acid differences between two variants of DEN1-NS1 (variant 1: NAD1 and PD1-1, variant 2: PD1-2 and PD1-3) within 352 amino acids (Supplementary Figure S11 and Figure 6A).

### UB-DNA aptamer generation targeting DEN1-NS1 variant 2

To evaluate the high specificity of these UB-DNA aptamers, we performed ExSELEX targeting DEN1-NS1 sub-serotype variant 2 derived from PD1-2, which was not detected by AptD1. The target DEN1-NS1 variant 2 was prepared by the conventional CHO cell expression system. In this selection, we also used the patient clinical sample infected by DENV as the target. We performed seven rounds of ExSELEX, in which we used the prepared variant 2 protein in the first, second, third and sixth rounds (Supplementary Table S3). For the other rounds, we directly used the DENV patient's serum (PD1-13) containing DEN1-NS1 with the same sequence as variant 2, instead of the prepared variant 2 protein. In the selection step, DNA libraries were incubated with the serum, and the DNA−target protein complexes were captured using the immobilized Ab#D06 antibody. After seven rounds of selection, we isolated a dominant sequence family (19D1F1) that binds specifically to the target DEN1-NS1 variant 2 from the enriched library (Supplementary Figures S12 and S13). In the EMSA, we observed the band of the aptamer−target protein complex even in the presence of 2 M urea in the gel and confirmed the specificity to DEN1-NS1 variant 2 and the dependency on the Ds bases for the tight binding (Supplementary Figure S12B).

Finally, we optimized and extended the sequence with the biotinylated mini-hairpin DNA, generating the AptD1b (19D1F1-3) aptamer with a *K*_D_ of 27 pM (Figure 6 and Supplementary Figures S13B and S14). The binding experiments using a series of deletion variants of the aptamer (Supplementary Figure S13B) revealed that the relatively long length (∼78-mer) of the AptD1b aptamer was required for the tight binding with DEN1-NS1 variant 2, and several types of secondary structures were presumed (Supplementary Figure S13C). The terminal stem region involves the 5′- and 3′-primer sequences in the library, and thus might contribute to the complex stability by the interactions of the phosphates in this region with DEN1-NS1 variant 2, rather than by sequence-specific interactions.

The ELISA format using the AptD1b and Ab#D06 sandwich pair efficiently detected only the DEN1-NS1 variant 2 in the patient sera of PD1-2 and PD1-3, but not those of PD1-1 and all four DEN-NS1 serotypes purchased from The Native Antigen Company (Figure 6C). Thus, these two Ds-DNA aptamers, AptD1 and AptD1b, can recognize either sub-serotype variant 1 or variant 2 of DEN1-NS1 with 10 amino-acid differences in 352 amino acids.

### Highly specific DEN1-NS1 variant detection in clinical samples by ELISA using AptD1 and AptD1b

**Figure 7.**
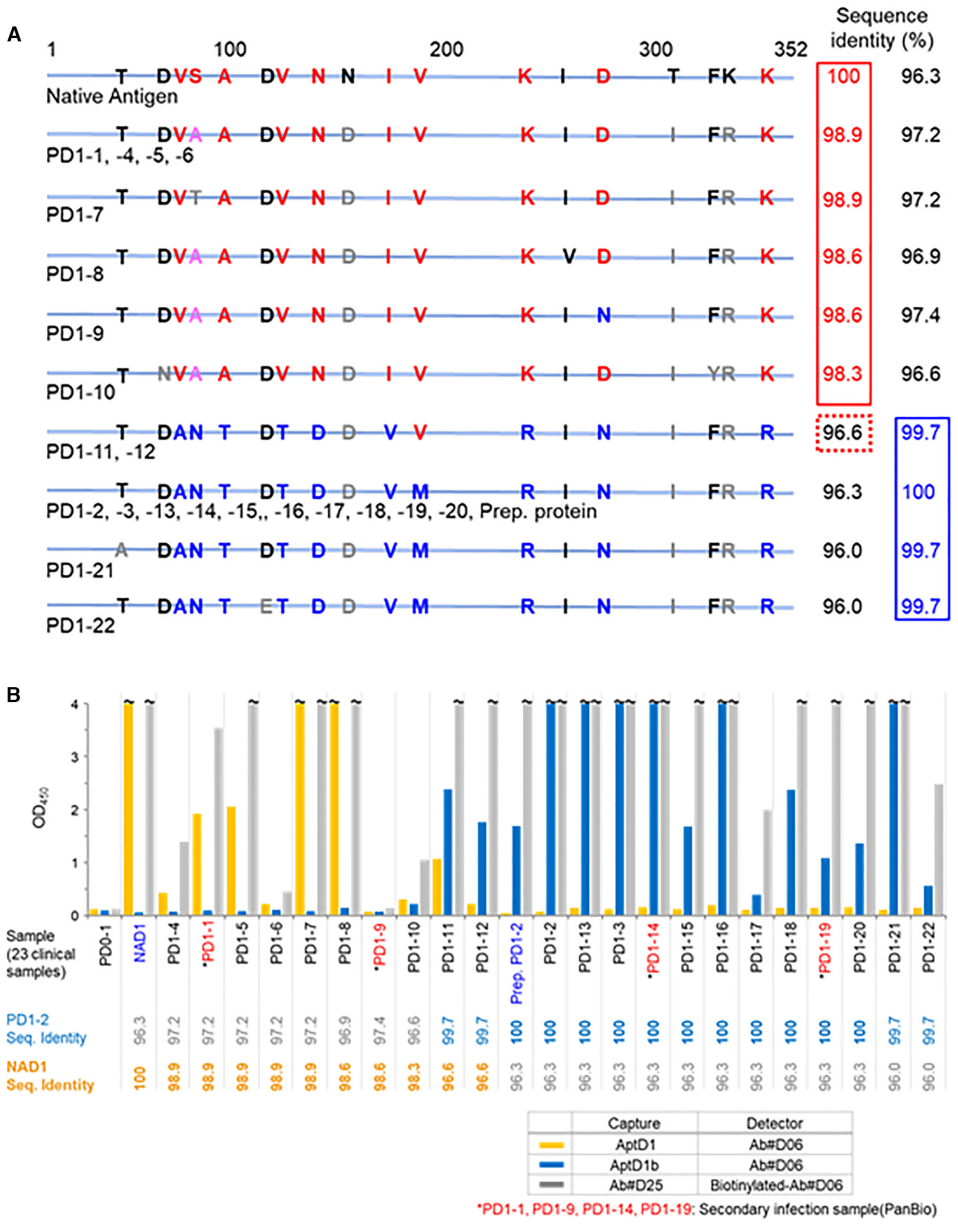
DEN1-NS1 variant detection in 22 Singaporean clinical samples by the aptamer−antibody ELISA formats. (**A**) Differences in the amino acid sequences of DEN1-NS1 proteins derived from the 22 clinical samples (PD1-1 through PD1-22) in Singapore and the original DEN1-NS1 target from The Native Antigen Company (Native Antigen / NAD1). The sequence identities (%) are presented as compared to NAD1 (left column) and PD1-2/1-3 (right column). (**B**) The DEN-NS1 variant detection by our ELISA formats, using aptamer (AptD1 or AptD1b) and antibody (Ab#D06) pairs and an antibody (Ab#D25) and antibody (Ab#D06) pair. Alere's LFA (Panbio Dengue Duo Cassette for IgM and IgG) assigned PD1-1, PD1-9, PD1-14 and PD1-19 as the secondary DENV infected samples. The amino-acid sequence identities, Seq. Identity (%), with DEN1-NS1 in PD1-2 and with NAD are shown at the bottom of the graph.

The two Ds-DNA aptamers, AptD1 and AptD1b, sensitively recognized DEN1-NS1 variant 1 and variant 2, respectively. To assess the sensitivity and specificity of the ELISA format using these two aptamers, we tested 22 Singaporean clinical samples of DENV serotype 1 infections, obtained within 3−5 days after fever onset (PD1-1 through PD1-22) (Supplementary Table S1), as well as control human serum (PD0-1) collected from a Japanese person who has never been infected with DENV. The Dengue RT-qPCR test kit (Fast Track Diagnostics) did not identify the sequence differences between variants 1 and 2 in the 22 samples. We determined the DEN1-NS1 sequences of all of the clinical samples, by RT-PCR and sequencing of the DEN-NS1-encoded mRNA extracted from each sample (Figure 7A and Supplementary Figure S15).

Most of these patient samples were assigned into two families, DEN1-NS1 variant 1 or variant 2, by their sequence identities from viral RNA sequencing. The sequences of DEN1-NS1 (PD1-4 through PD1-10) in seven patients have high sequence identity (≥98.3%) with that of variant 1 (NAD1 and PD1-1). The sequences of the other 10 patient samples (PD1-13 through PD1-22) are quite similar (≥99.7%) to those of variant 2 (PD1-2 and PD1-3), and the sequence identities between these variant 2 sequences and the typical variant 1 (PD1-1) are ≤96.3%. The sequences of two other samples of PD1-11 and PD1-12 are a mixture of variants 1 and 2.

As compared to the signals of PD0-1, our ELISA format detected DEN1-NS1 in 21 of the 22 patient samples, except PD1-9 (Figure 7B). The two UB-DNA aptamers, AptD1 and AptD1b, clearly identified either DEN1-NS1 variant 1 or 2 in the 21 patient samples (yellow bars for variant 1 and blue bars for variant 2 in Figure 7B). The ELISA using the antibody−antibody sandwich system with Ab#D06 and Ab#D25 detected DEN-NS1 proteins in all of the samples, except PD1-9 (gray bars in Figure 7B). Interestingly, AptD1 and AptD1b both detected DEN1-NS1 of PD1-11, in one of two samples (PD1-11 and PD1-12) with the mixed sequences of variants 1 and 2. Since AptD1b detected DEN1-NS1 of PD1-11 and PD1-12 with higher sensitivities than AptD1, our ELISA format classified these samples as variant 2. Actually, nine of the ten amino acids of these DEN1-NS1 proteins in PD1-11 and PD1-12 are the same as those in variant 2. In the tests of the 22 samples (PD1-1 through PD1-22), the DEN1-NS1 proteins in the 21 samples were assigned to either variant 1 or variant 2. These results are completely consistent with the sequence analyses of these clinical samples.

The aptamer−antibody and antibody−antibody pair ELISA formats could not detect DEN1-NS1 in PD1-9. This clinical sample contains IgGs to DEN1-NS1 due to the secondary DENV infection, and thus the patient's anti-DEN-NS1 IgGs competitively reduce the detection sensitivity of the ELISA formats. Serological tests using the anti-DEN-NS1 IgM and IgG detection kits (Panbio) indicated the possibility of secondary DENV infections in the PD1-1, PD1-9, PD1-14 and PD1-19 patients. The PD1-9 patient was previously infected with DENV and their sample contained anti-DEN1-NS1 IgGs even in the early stage (within four days after fever onset), preventing the DEN-NS1 detection by the aptamer−antibody and antibody−antibody ELISA systems. The other samples with secondary infection (PD1-1, PD1-14 and PD1-19) contained IgGs against other DEN-NS1 serotypes (data not shown), and thus the ELISA formats detected each DEN1-NS1 variant. From these observations, we conceived an idea to develop a dengue serotype-specific IgG detection method by competitive ELISA format using the UB-DNA aptamers (53).

Although the sensitivities using the aptamer−antibody sandwich are relatively lower than those using the antibody−antibody sandwich (Figure 7B), this arises from the different detection systems between the two sandwich methods. For the antibody−antibody sandwich method, we used biotinylated Ab#D06 as the detector agent and a streptavidin-HRP conjugated secondary detector antibody. Since multiple biotinylations of Ab#D06 occurred, multiple secondary detectors (streptavidin-HRP-conjugated antibodies) were bound to Ab#D06. In contrast, for the aptamer−antibody sandwich, we employed an anti-rabbit IgG HRP conjugate as the secondary detector, which binds to the Fc region of Ab#D06 with the 1-to-1 complex. This is because we used the biotinylated aptamers as the capture agent for the immobilization on the streptavidin-coated plate in the aptamer−antibody sandwich. Thus, the antibody−antibody sandwich method with the binding of multiple secondary detector antibodies exhibited higher sensitivity than that of the current aptamer−antibody sandwich method. The sensitivity of the aptamer−antibody sandwich method could be further improved by changing the aptamer immobilization method (10).

## DISCUSSION

We have presented a series of high-affinity and high-specificity UB-DNA aptamers targeting each DEN-NS1 variant. Using these UB-DNA aptamers, the exceptionally sensitive ELISA format enables the detection of each DEN-NS1 sub-serotype variant beyond the four-serotype DENV identification. Our study and results provide several insights into (1) high-affinity aptamer generation begetting high specificity, (2) six-letter DNA aptamer generation, and (3) diagnostics using UB-DNA aptamers for infectious diseases.

### High-affinity aptamer generation begetting high specificity

DNA aptamers are generated by affinity selection to targets from DNA libraries through the SELEX procedure. In particular, we employed a washing buffer containing 2−3 M urea to remove the weaker binders from the DNA-target complexes at later rounds of selection, and then generated a series of UB-DNA aptamers with 27−182 pM *K*_D_ values. These high-affinity UB-DNA aptamers bind specifically to the target DEN-NS1 families with ≥96.9% amino acid sequence identities. In other words, these aptamers recognize ∼10 amino acid differences between sub-serotypes variant 1 and variant 2 of DEN1-NS1 (352 amino acids).

The presence of only two Ds bases in AptD1 and AptD1b significantly increases the affinities and specificities to each target. Thus, the differences of some nonpolar amino acid residues between DEN1-NS1 variants 1 and 2 might affect the aptamer specificity, such as at positions 93 (Val and Ala in variants 1 and 2, respectively), 98 (Ala and Thr), 128 (Val and Thr) and 178 (Val and Met) (Figure 7 and Supplementary Figures S15 and S16). The ELISA data using PD1-11 and PD1-12 detection by AptD1 and AptD1b suggest the importance of position 178 (Val and Met) for the aptamer recognition. However, if only these two Ds bases and/or one Px base recognize each target DEN-NS1 protein, then it seems rather difficult to explain the high specificities and affinities of these aptamers to each serotype or variant. One of the plausible reasons is that the UB bases are located quite close to the appropriate amino acid residues (nonpolar amino acids for Ds and polar amino acids for Px), as supported by fitting the entire tertiary structure of each aptamer to the shape of its target protein. This is why most of the UB-DNA aptamers that we generated form stable secondary structures, including the 5′- and 3′-terminal stem structures (Figure 2 and Supplementary Figure S13C) (17,21). Furthermore, the sequences of the aptamers are completely different, and thus each aptamer might form unique tertiary structures, such as the G-quadruplex of AptD2-1, to fit each target structure. In addition to the UBs, the interactions of many natural base residues in the aptamers with the amino acid residues of the targets reinforce the specificities and affinities. The high specificity of unique structures of aptamers is also observed in conventional aptamers: pseudoknot natural-base RNA aptamers recognize one-point mutation of the target, HIV-1 RT, but non-pseudoknot RNA aptamers exhibit broad-spectrum for the binding (29). Accordingly, UB-DNA aptamers generated by affinity selection naturally exhibit high specificities to their targets. Other high-affinity UB-DNA aptamers have also exhibited enhanced specificities (16,21,22,49). However, DEN-NS1 forms a hexameric complex, and the structure of the aptamer-bound form and the mechanism of sandwich formation among the aptamer–target–antibody complexes remain enigmatic. Thus, further structural analyses are required to clarify the exact interaction of these aptamers with each DEN-NS1 serotype or variant.

Through this research, we optimized the methods and conditions of ExSELEX. In this study, three ExSELEX methods (ExSELEX-1 to ExSELEX-3 in Supplementary Table S2) were first employed for the generation of aptamers targeting each serotype of the commercially available DEN-NS1 proteins conjugated with His-Tags. Based on these results, we optimized the method as ExSELEX-4 (Supplementary Table S3), using the targets of the prepared DEN1-NS1 variant 2 protein or in the clinical samples from the patients infected by DENV serotype 1 variant 2. In ExSELEX-4, to shift from the simple to more complex selection conditions, we employed multiple selection platforms, including the His-Tag separation of the DNA−protein complexes using Ni-NTA magnetic beads, the complex separation using the anti-DEN-NS1 antibody immobilized on the plates, and the isolation of the DNA that binds to DEN-NS1 in the clinical samples, using the antibody. We employed the prepared proteins as the targets in the early rounds of selection and the clinical samples in the later rounds, because the DEN1-NS1 concentrations in the clinical samples are very low (i.e., 30 ng/mL DEN-NS1 corresponds to 100 pM in the hexamer form). The combination of these platforms from simple to complex conditions effectively functioned for the aptamer generation toward practical use. To increase the selection pressure, the concentrations of the library and the target protein were gradually reduced throughout the entire rounds of the selection. In addition, in the later rounds of selection, human serum was also added into the binding buffer. Furthermore, the washing conditions with 2–3 M urea are useful to select aptamer candidates with high affinities. These conditions were determined based on the qPCR data in each selection round, as mentioned in the Results section.

### Six-letter DNA aptamer generation

We have presented the serendipitous development of six-letter DNA aptamers (AptD2) containing Px in addition to the intended Ds bases that selectively target one of the DEN2-NS1 sub-serotype variants. AptD2 exhibited the highest affinity, as compared to the other aptamer candidates containing only Ds bases, targeting this DEN2-NS1 variant, indicating the importance of the sixth UB for developing high-affinity aptamers to some targets. Since the replacement of the 2-nitropyrrole moiety of Px with the pyrrole-2-carbaldehyde of Pa retains the aptamer's high affinity, the diol residue of Px is likely the main contributor to the affinity. As a strong proton donor or acceptor group, the diol residue, which does not exist in the 20 standard amino acid residues, might uniquely interact with some polar amino acid residues of DEN2-NS1.

Furthermore, AptD2 contains several G-tract regions, suggesting the possible G-quadruplex formation (Figure 2). Using the G-to-A scanning method, we identified the four G-tracts that could be involved in the G-quadruplex formation. Although we still do not know the actual topology of the G-quadruplex, the antiparallel topologies (54,55) are likely when considering the terminal stem and the short loop region around the Pa base (Supplementary Figure S17). In these antiparallel topologies, the essential bases including Ds11, Ds23 and Pa35, as well as G15 and G48 in AptD2-1, are located on the same interface in the antiparallel structures, and thus could interact with DEN2-NS1. Generally, only the small loop regions in G-quadruplex aptamers interact with target proteins, and thus the increase in the chemical diversity, especially in the small loop regions, might be important for the tight and specific binding to targets. Therefore, the expansion of the chemical diversity with the combination of the hydrophobic Ds and hydrophilic Px bases as fifth and sixth letters could further improve the aptamer affinity and specificity, as well as increase the success rate of aptamer generation.

Benner's team also reported six-letter DNA aptamer generation methods using their hydrogen-bonded UBP (Z and P), in which UBs with the similar hydrophilic physicochemical properties to those of natural bases moderately improved the DNA aptamer affinity (4,56,57). Thus, in addition to the differences between our UBs and the natural bases, the unique properties of Ds and Px could greatly enhance the abilities of UB-DNA aptamers. Our results encourage us to develop six-letter DNA aptamer generation methods. We confirmed that AptD2 did not bind to another DEN2-NS1 sub-serotype, which is the main variant included in the Singaporean patient samples, PD2-2/PD2-3 (data not shown), and UB-DNA aptamer generation targeting this DEN2-NS1 sub-serotype variant is in progress.

### Diagnostics using UB-DNA aptamers for infectious diseases

The ELISA format using the UB-aptamer and antibody sandwich pair provides a highly specific diagnostic tool. As reported previously, sensitive ELISA formats require high-affinity ligands with *K*_D_ values lower than hundreds of pM (10). Thus, we generated a series of UB-DNA aptamers (*K*_D_ = 27−182 pM) for the DEN-NS1 detection. Our ELISA method using UB-DNA aptamers detects only a few amino acid changes in the target proteins during the early phase of DENV infection.

Our highly specific DEN1-NS1 variant detection results demonstrate the tracing and classifying methods of virus mutations, which are important for the rapid investigation of the viral mutation effects on the clinical conditions in endemic, epidemic, and pandemic forms. To this end, a rapid aptamer generation (within 2−4 weeks) targeting each viral mutant will be essential. Our development of the AptD1 and AptD1b aptamers demonstrates this possibility, using patient sera instead of target proteins for ExSELEX. Capturing the viral biomarkers in the patients’ blood samples, using immobilized antibodies with broad specificities, allows for rapid high-specificity aptamer generation. This methodology using UB-DNA aptamers could be applied to other detection formats (58), and provide a wide range of infectious diseases and biomarker detection systems to identify target variants and mutants. The high affinities and superior specificities of UB-DNA aptamers could greatly improve the sensitivities and specificities of the current diagnostic methods.

Furthermore, this method can be used as a competitive ELISA format for a serological test of the DENV serotype identification, by detecting the competition with anti-DEN-NS1 IgGs (53). We found that the binding of these UB-DNA aptamers to each DEN-NS1 is only competed by IgGs with high affinity to DEN-NS1, which would in turn enable the detection of serotype-specific IgGs. In addition, anti-DEN-NS1 IgMs do not inhibit the UB-DNA aptamer binding to DEN-NS1. This competitive ELISA format is useful for the IgG serotype identification of the later phase of dengue infection and past infection, as well as the evaluation and development of dengue vaccine treatments.

## Supplementary Material

gkab515_Supplemental_File
