# Supplementary material for: High-affinity five/six-letter DNA aptamers with superior specificity enabling the detection of dengue NS1 protein variants beyond the serotype identification
Source: Nucleic Acids Res. 2021 Jun 25;49(20):11407–24. doi: 10.1093/nar/gkab515 (PMC8599795; doi:10.1093/nar/gkab515)
Supplement: gkab515_Supplemental_File [file gkab515_supplemental_file.pdf]

Supplementary Information

High-affinity five/six-letter DNA aptamers with superior specificity enabling the detection of dengue NS1 protein variants beyond the serotype identification

Ken-ichiro Matsunaga[1], Michiko Kimoto[1], Vanessa Weixun Lim[2], Hui Pen Tan[1], Yu Qian Wong[1], William Sun[1,3], Shawn Vasoo[2,4,5], Yee Sin Leo[2,4,5,6], and Ichiro Hirao[1,]*

[1]Institute of Bioengineering and Bioimaging, 31 Biopolis Way, The Nanos, #07-01, Singapore 138669, Singapore,
[2]National Centre for Infectious Diseases, 16 Jalan Tan Tock Seng, Singapore 308442, Singapore
[3]Department of Physiology, Yong Loo Lin School of Medicine, National University of Singapore, Singapore 117593, Singapore,
[4]Department of Infectious Diseases, Tan Tock Seng Hospital, 11 Jalan Tan Tock Seng, Singapore 308433, Singapore,
[5]Lee Kong Chian School of Medicine, Nanyang Technological University, 59 Nanyang Dr, Experimental Medicine Building, Singapore 636921, Singapore,
[6]Saw Swee Hock School of Public Health, National University of Singapore, 12 Science Drive 2, #10-01, Singapore 117549, Singapore.
* To whom correspondence should be addressed. Tel: +65-6824-7104; Fax: +65-6478-9083; Email: ichiro@ibb.a-star.edu.sg

Supplementary Table S1: Patient sample information.
Supplementary Table S2: ExSELEX conditions targeting each DEN-NS1 serotype.
Supplementary Table S3: ExSELEX conditions targeting DEN1-NS1 variant 2.
Supplementary Table S4: Summary of the sequence reads and clustering information for each enriched library.
Supplementary Table S5: Sequences of anti-DEN-NS1 DNA aptamer candidates.
Supplementary Fig. S1: Sequences of four serotypes of DEN-NS1 proteins used in ExSELEX.
Supplementary Fig. S2: The kinetic binding parameters, dissociation constant (K_D) and association and dissociation rates (k_{on} and k_{off}) of rabbit monoclonal antibodies, Ab#D06 and Ab#D25.
Supplementary Fig. S3: Quantitative PCR analysis of ExSELEX-1.
Supplementary Fig. S4: Binding analysis of DNA libraries by electrophoresis gel-mobility shift assays (EMSA).
Supplementary Fig. S5: Alignment of the random-region DNA sequences obtained by the three ExSELEX procedures, targeting DEN1-NS1.
Supplementary Fig. S6: Alignment of the random-region DNA sequences obtained by the three ExSELEX procedures, targeting DEN2-NS1.
Supplementary Fig. S7: Alignment of the random-region DNA sequences obtained by the three ExSELEX procedures, targeting DEN3-NS1.
Supplementary Fig. S8: Alignment of the random-region DNA sequences obtained by the three ExSELEX procedures, targeting DEN4-NS1.
Supplementary Fig. S9: Confirmation of the presence of diol-Px in the selected clone family, D2-1.
Supplementary Fig. S10: Limit of detection (LOD) and limit of quantification (LOQ) targeting each dengue serotype NS1 by a sandwich-type ELISA.
Supplementary Fig. S11: Differences in the amino acid sequences of DEN-NS1 proteins in the clinical samples.
Supplementary Fig. S12: Binding analysis of the enriched DNA library and 19D1F1 to DEN-NS1 variant 2 by EMSA.
Supplementary Fig. S13: The UB-DNA aptamer generation targeting DEN1-NS1 variant 2.
Supplementary Fig. S14: Binding analysis of UB-DNA aptamers, 19D1F1-3 (AptD1b) and 19D1F1 (isolate), to each target by a Biacore T200 SPR system at 25°C.
Supplementary Fig. S15: Differences in the amino acid sequences of DEN1-NS1 proteins in the clinical samples, PD1-1 through PD1-21.
Supplementary Fig. S16: NS1 sequence variations of dengue serotype 1 clinical samples.
Supplementary Fig. S17: Possible topologies of the G-quadruplex of AptD2-1.

Supplementary Table S1. Patient sample information. The recruited patients were tested and confirmed as DENV NS1 positive by routine hospital diagnostics, using the SD BIOLINE NS1 Ag rapid test, and had fevers within 3–5 days from illness onset. The DENV infection was confirmed by an RT-qPCR analysis of the samples, and the dengue serotypes were determined by an FTD dengue differentiation RT-qPCR test from Fast Track Diagnostics, using a Bio-Rad CFX96 PCR system. The samples in Figure 5 are indicated with asterisks.

Patient Sample		Fever day (from illness onset)	Serotype (RT-qPCR)	SD BIOLINE Dengue NS1 Ag rapid test
PD1-1*	Serum	5	D1	+
PD1-2*	Serum	4	D1	+
PD1-3*	Serum	4	D1	+
PD1-4	Serum	5	D1	+
PD1-5	Serum	4	D1	+
PD1-6	Serum	3	D1	+
PD1-7	Serum	4	D1	+
PD1-8	Serum	4	D1	+
PD1-9	Serum	4	D1	+
PD1-10	Serum	5	D1	+
PD1-11	Serum	5	D1	+
PD1-12	Serum	3	D1	+
PD1-13	Serum	5	D1	+
PD1-14	Serum	4	D1	+
PD1-15	Serum	3	D1	+
PD1-16	Serum	4	D1	+
PD1-17	Serum	4	D1	+
PD1-18	Serum	5	D1	+
PD1-19	Serum	4	D1	+
PD1-20	Serum	5	D1	+
PD1-21	Serum	5	D1	+
PD1-22	Serum	3	D1	+
PD2-1*	Plasma	3	D2	+
PD2-2*	Serum	3	D2	+
PD2-3*	Serum	5	D2	+
PD3-1*	Serum	5	D3	+
PD3-2*	Serum	4	D3	+
PD3-3*	Serum	4	D3	+
PD4-1*	Serum	3	D4	+

Supplementary Table S2. ExSELEX conditions targeting each DEN-NS1 serotype. We performed ExSELEX targeting each serotype of DEN-NS1 proteins, as follows: DEN1-NS1 (D1), DEN2-NS1 (D2), DEN3-NS1 (D3), and DEN4-NS1 (D4) in the PCR cycles column. To increase the stringency of the selection conditions, we added human serum (HS) and/or BSA to the binding buffer (additives) and urea in the washing buffer in later rounds.

ExSELEX-1

Round	Method	DNA [nM]	Target [nM]	Volume [mL]	Additives	Binding		Washing	Counter Selection	PCR cycles			
						Buffer	Time (min)			D1	D2	D3	D4
1	A	500	5	8	-	BB1	60	BB1 ×3	-	18	18	18	18
2	A	100	5	1	-	BB1	30	BB1 ×5	Pre	10	12	10	12
3	B	20	4	0.2	0.1% BSA	BB1	30	WB ×5	Pre	25	29	28	23
4	B	5	4	0.2	0.1% BSA, 5% HS	BB1	30	WB ×10	Pre, Post	14	18	22	22
5	B	5	0.4	0.2	0.1% BSA,10% HS	BB1	30	WB ×25	Pre, Post	15	17	14	17
6	B	5	0.4	0.2	0.1% BSA, 20% HS	BB1	30	WB (+ 20% HS) ×3, WB ×5	Pre, Post	12	15	12	14
7	B	1	0.4	0.3	0.1% BSA, 50% HS	BB1	30	WB (+ 50% HS) ×3, WB ×10	Pre, Post	13	16	16	18
8	B	1	0.04	0.6	0.1% BSA, 50% HS	BB1	30	WB (+ 50% HS) ×3, WB ×10	Pre, Post	20	21	23	23
9	C	1	0.167	1	0.1% BSA	BB1	30	WB (+ 2M urea) ×3, WB ×2	Pre, Post	28	22	24	19
									Total	155	168	167	166

ExSELEX-2

Round	Method	DNA [nM]	Target [nM]	Volume [mL]	Additives	Binding		Washing	Counter Selection	PCR cycles			
						Buffer	Time (min)			D1	D2	D3	D4
1	C	500	5	8	-	BB1	60	BB1 ×3	-	20	20	20	20
2	C	100	5	1	-	BB1	30	BB1 ×5	Pre	22	22	19	20
3	B	50	2.5	0.4	0.1% BSA, 10% HS	BB1	30	WB ×5	Pre	15	21	25	21
4	B	10	1	0.4	0.1% BSA, 50% HS	BB1	30	WB ×10	Pre, Post	20	24	25	19
5	C	3	1	1	0.1% BSA	BB1	15	BB1 (+ 3 M urea) ×3, BB1 ×2	Pre, Post	26	20	27	19
6	B	3	1	0.4	0.1% BSA, 50% HS	BB1	30	WB ×10	Pre, Post	18	20	24	16
7	C	3	1	1	0.1% BSA	BB1	15	BB1 (+ 3 M urea) ×3, BB1 ×2	Pre,Post	24	18	27	18
8	B	1	0.5	0.4	0.1% BSA, 50% HS	BB1	30	WB ×10	Pre, Post	23	23	25	21
9	B	1	0.5	0.4	0.1% BSA,50% HS	BB1	30	WB ×20	Pre, Post	23	24	27	21
10	D	20	10	0.02	-	BB1	30	-	-	12	12	12	12
									Total	203	204	231	187

ExSELEX-3

Round	Method	DNA [nM]	Target [nM]	Volume [mL]	Additives	Binding		Washing	Counter Selection	PCR cycles		
						Buffer	Time (min)			D1	D2	D3
1	B	2500	5	0.8	0.1% BSA, 10% HS	BB2	30	WB ×3	-	21	22	20
2	B	250	5	0.3	0.1% BSA, 10% HS	BB2	30	WB ×5	Pre	15	20	21
3	B	50	5	0.3	0.1% BSA, 20% HS	BB2	30	WB ×5	Pre	15	15	15
4	B	5	1	0.3	0.1% BSA, 45% HS	BB2	30	WB (+2 M urea) ×3, WB ×2	Pre	24	27	23
5	B	1	0.2	0.3	0.1% BSA, 45% HS	BB2	10	WB (+2 M urea) ×3, WB ×2	Pre	24	25	28
6	B	1	0.2	0.3	0.1% BSA, 45% HS	BB2	5	WB (+50% HS) ×2, WB (+ 2 M urea) ×2, WB ×2	Pre	23	20	27
7	B	0.5	0.2	0.3	0.1% BSA, 45% HS	BB2	5	WB (+50% HS) ×3, WB (+ 3 M urea) ×3, WB ×3	Pre	25	25	29
									Total	147	154	163

Separation of DNA-target complexes (Method):
A: Ultrafiltration (Amicon Ultra-100kDa)
B: Sandwich (Capture with mAb#D06, in 96-well plates)
C: Complex immobilization (Dynabeads™ His-Tag Isolation and Pulldown)
D: Separation by gel-mobility shift [4% PAGE (29:1 acrylamide -bisacrylamide) supplemented with 5% glycerol and 2 M urea]

Buffers:
BB1: 20 mM Tris-HCl (pH 7.5), 150 mM NaCl, 1 mM $MgCl_2$, 2.7 mM KCl, 0.005% Nonidet-P40
BB2: 20 mM Tris-HCl (pH 7.5), 150 mM NaCl, 1 mM $MgCl_2$, 2.7 mM KCl, 2% Tween 20
WB: 20 mM Tris-HCl (pH 7.5), 150 mM NaCl, 1 mM $MgCl_2$, 2.7 mM KCl, 0.05% Tween 20

Supplementary Table S3. ExSELEX conditions targeting DEN1-NS1 variant 2. We performed ExSELEX targeting recombinant DEN1-NS1 variant 2 protein and clinical serum (PD1-13, the antigen NS1 sequence was the same as that of variant 2). To increase the stringency of the selection conditions, we added human serum (HS) and BSA to the binding buffer (additives) and urea in the washing buffer in later rounds.

ExSELEX-4

Round	Selection Method	DNA [nM]	Target		Volume [ml]	Aditives	Binding		Washing	Counter Selection	PCR Cycles
			DEN1-NS variant 2 [nM]	Clinical Serum [µl]			Buffer	Time(min)			
1	C	500	2.5	-	0.8	-	BB1	60	WB1 × 3	-	20
2	C	200	2.5	-	0.3	-	BB1	30	WB1 × 5	Pre	20
3	B	50	2.5	-	0.4	0.1% BSA	BB2	30	WB2 × 6	Pre, Post	22
4	B	10	-	20	0.4	0.1% BSA, 10% HS	BB2	30	WB2 × 6	Pre, Post	15
5	B	10	-	10	0.4	0.1% BSA, 10% HS	BB2	30	WB2 (+ 3M Urea) ×10	Pre, Post	28
6	B	3	1	-	0.4	0.1% BSA, 10% HS	BB2	30	WB2 (+ 3M Urea) ×10	Pre, Post	22
7	B	3	-	5	0.4	0.1% BSA, 10% HS	BB2	30	WB2 (+ 3M Urea) ×10	Pre, Post	28
										Total	155

Separation of DNA-Target Complexes (Method) :
B: Sandwich (Capture with mAb#D06, in 96-well plates)
C: Complex immobilization (Dynabeads™ His-Tag Isolation & Pulldown)

Buffers :
BB1: 20 mM Tris-HCl, pH 7.5, 150 mM NaCl, 1 mM $MgCl_2$, 2.7 mM KCl, 5 mM Imidazole, 0.005% Nonidet-P40
BB2: 20 mM Tris-HCl, pH 7.5, 150 mM NaCl, 1 mM $MgCl_2$, 2.7 mM KCl, 5 mM Imidazole, 0.05% Tween 20
WB1: 20 mM Tris-HCl, pH 7.5, 150 mM NaCl, 1 mM $MgCl_2$, 2.7 mM KCl, 0.005% Nonidet P-40
WB2: 20 mM Tris-HCl, pH 7.5, 150 mM NaCl, 1 mM $MgCl_2$, 2.7 mM KCl, 0.05% Tween 20

Supplementary Table S4: Summary of the sequence reads and clustering information for each enriched library. The unnatural bases in the DNA libraries were replaced with the natural bases by replacement PCR in the presence of dPa′TP (Pa′) or diol-dPxTP (Px), and the natural-base libraries were subjected to deep sequencing with an IonPGM system using the indicated chip types. “Total Read” corresponds to the sequencing reads after automated QC in the IonPGM Torrent Server, and “Extract” corresponds to the merged extracted reads after the primer sequencing trimming criteria: 5′-(full sequence of the forward/reverse primer)-[42 bases]-(complementary sequence of the last six bases of the reverse/forward primer)-3′. The merged extracted sequences were then clustered based on the sequence similarities, using in-house Perl scripts. The clustering criteria were to cluster a sequence into the same family if the mismatch between the sequence and the top sequence is less than six, and the cut-off of the family was set to 20 reads. It should be noted that the same family mainly comes from the same clone sequence, but harboring different natural base patterns at the unnatural base positions and with sequencing errors, besides similar clones with some mutations, which belong to a usual ‘family” in the case of natural-base aptamers. We mainly focused on the top-ranked families for further analysis, since the enriched libraries exhibited clear binding to each target in gel-mobility shift assays (EMSA).

ExSELEX	Round	IonPGM					Total clustered family number		Selected families for further analysis (Pa')		
		Ion PGM chip	Total Read		Extracted						
			Pa'	Px	Pa'	Px	Pa'	Px	Family number	Selection criteria	Covered population (Pa')
ExSELEX-1-D1	9	314	103,693	145,765	39,389	53,596	89	93	17	>500 reads (1.3%)	63.1%
ExSELEX-2-D1	10	314	103,319	106,064	56,393	56,411	26	21	6	>500 reads (0.9%)	94.3%
ExSELEX-3-D1	7	316	496,332	465,076	269,007	288,202	294	275	14	>2% populations	60.9%
ExSELEX-1-D2	9	314	95,511	131,017	50,663	75,426	25	36	10	>500 reads (1%)	93.8%
ExSELEX-2-D2	10	314	90,880	93,077	66,877	57,383	11	12	8	>500 reads (0.7%)	98.5%
ExSELEX-3-D2	7	316	425,100	542,125	293,912	362,713	93	121	5	>2% populations	82.4%
ExSELEX-1-D3	9	314	67,232	117,162	45,611	79,771	12	13	4	>500 reads (1.1%)	96.8%
ExSELEX-2-D3	10	314	90,946	70,574	64,544	46,669	10	4	2	>500 reads (0.8%)	96.8%
ExSELEX-3-D3	7	316	509,598	432,128	211,895	158,682	61	45	7	>2% populations	88.4%
ExSELEX-1-D4	9	314	58,514	130,996	39,094	88,975	16	15	8	>500 reads (1.3%)	97.0%
ExSELEX-2-D4	10	314	104,741	87,325	43,797	38,672	31	29	10	>500 reads (1.1%)	88.5%
ExSELEX-4-D1	7	314	99,705	95,668	43,385	33,387	9	10	2	>500 reads (1.2%)	95.1%

Supplementary Table S5: Sequences of anti-DEN-NS1 DNA aptamer candidates. The oligonucleotide sequences used for the binding analyses against each target DEN-NS1 are summarized with the results of the electrophoresis gel-mobility shift assay (EMSA) and the surface plasmon resonance (SPR) analysis. The additional complementary sequences are underlined, and the changed sequences are shown in pink. The sequences in the constant primer regions are indicated in lower-case letters. The oligonucleotides containing a mini-hairpin sequence, CGCG-(Biotin-T)-AGCG, at the 3'-terminus have an additional "h" in the aptamer candidate names. In the SPR analysis with 20 nM of each dengue NS1 protein, "specific" means that the oligonucleotide only bound to the target serotype DEN-NS1, and not to the other serotype DEN-NS1, while "less-specific" means the oligonucleotide exhibited binding to not only the target serotype NS1 but also to some of the other serotype NS1 proteins. The chemical structures of the unnatural bases, diol-Px (Px) and diol-Pa (Pa), are illustrated at the bottom of the table.

Name	EMSA	SPR		Sequence (5'- to -3': L = Biotin-dT, x = dDs)
**D1-1-78**	**(++) +++**	**K_D = 132 pM**	**specific**	LgatatggtctactgTGTGAxGTCCTACAATGGACTGGTGTxCTCGGxATGGCCATTgacaagcggagtagttagacc
**D1-1-42h**	**+++**	**K_D = 197 pM**	**specific**	CAGACGGACTGGTGTxCTCGGxATGGCCGTCTGCGCGLAGCG
**D1-1-48h (AptD1)**	**+++**	**K_D = 182 pM**	**specific**	CCCCAGACGGACTGGTGTxCTCGGxATGGCCGTCTGGGGCGCGLAGCG
**Cont-D1-1-48h**	**-**	**K_D = 1.3 μM**		CCCCAGACGGACTGGTGTACTCGGAATGGCCGTCTGGGGCGCGLAGCG
D1-2-78	(+)			LgatatggtctactgAGGAGCGCATGTCGAGATACCAACCxCCATCCAATCxTTCTTgacaagcggagtagttagacc
D1-3-78	(+++) +++	K_D = 55 pM	non-specific	LgatatggtctactgACGCCGGGGCCCGTAxTCAGACGTATACxCATCAGGGCACATgacaagcggagtagttagacc
D1-3-47	+++	K_D = 98 pM	non-specific	CGAGGCCCGTAxTCAGACGTATACxCATCAGGGCCTCGCGCGLAGCG
D1-4-78	(-)			LgatatggtctactgCCTGCACTGCCTCTGxAGCCCAACCxCCCATCCAATCGCTCAgacaagcggagtagttagacc
D1-5-61h	++			GGCAGCGCGTCGATTGxCCAATCTTAGCCAACCCAAAATTacaagcgCTgCCCGCGLAGCG
D1-6-47h	++			GCTGCCTxGTACCAACCCCCTCCAATCxATTAGGCAGCCGCGLAGCG
D1-7-51h	++			CGTGCGACGAxGTCCAACCAGTCCCAATCxACAAGTCGCACGCGCGLAGCG
**Name**	**EMSA**	**SPR**		**Sequence (5'- to -3': l = Biotin, L = Biotin-dT, x = dDs, d = Diol-dPa, y = Diol-dPx)**
**D2-1-78**	(-)			LgatatggtctactgGTCCGxCTGGGAACAAGxGGCGGGAGGGAxGGGTGTGGGTGCgacaagcggagtagttagacc
**D2-1-96(3Ds)**	**-**			tttcgcactccatgatatggtctactgGTCCGxCTGGGAACAAGxGGCGGGAGGGAxGGGTGTGGGTGCgacaagcggagtagttagaccgtcaaa
**D2-1d-97**	**++**	**K_D = 114 pM**		LtttcgcactccatgatatggtctactgGTCCGxCTGGGAACAAGxGGCGGGAGGGAdGGGTGTGGGTGCgacaagcggagtagttagaccgtcaaa
**D2-1y-96**	**+++**	**K_D = 41 pM**		ltttcgcactccatgatatggtctactgGTCCGxCTGGGAACAAGxGGCGGGAGGGAyGGGTGTGGGTGCgacaagcggagtagttagaccgtcaaa
**D2-1d-84**	**+**			LtttcgcactccatgatatggtctactgGTCCGxCTGGGAACAAGxGGCGGGAGGGAdGGGTGTGGGTGCgacaagcggagtag
**D2-1d-74**	**++**			LcatgatatggtctactgGTCCGxCTGGGAACAAGxGGCGGGAGGGAdGGGTGTGGGTGCgacaagcggagtag
**D2-1d-87h**	**++**	**K_D = 105 pM**	**specific**	GACggtctactgGTCCGxCTGGGAACAAGxGGCGGGAGGGAdGGGTGTGGGTGCgacaagcggagtagttagaccgtcCGCGLAGCG
**D2-1d-77h**	**++**			ggtctactgGTCCGxCTGGGAACAAGxGGCGGGAGGGAdGGGTGTGGGTGCgacaagcggagtagACCCGCGLAGCG
**D2-1d-72h (AptD2)**	**++/+++**	**K_D = 104 pM**	**specific**	GGctgGTCCGxCTGGGAACAAGxGGCGGGAGGGAdGGGTGTGGGTGCgacaagcggaCCagCCCGCGLAGCG
**Cont-D2-1d-72h**	**-**			GGctgGTCCGACTGGGAACAAGAGGCGGGAGGGATGGGTGTGGGTGCgacaagcggaCCagCCCGCGLAGCG
**D2-1d-61h**	**+++**			gGTCCGxCTGGGAACAAGxGGCGGGAGGGAdGGGTGTGGGTGCgacaagcggCGCGLAGCG
**D2-1d-51h**	**-**			gGTCCGxCTGGGAACAAGxGGCGGGAGGGAdGGGTGTGGGTGCGCGLAGCG
D2-2-78	(-)			LgatatggtctactgGAATAACAAGTCCGTGCxGTCGCCAATCCGTGxTCCAACCCCgacaagcggagtagttagacc
D2-2d-59h	+			GCGGTCCGTGCxGTCGCCAATCCGTGdTCCAACCCCgacaagcggaCCGCCGCGLAGCG
D2-3-78	(-)			LgatatggtctactgGTCTACATGCAACGCTTTCGxCCAACCCGTGxTCCAATCCCAgacaagcggagtagttagacc
D2-3d-52h	++			GCCCGCTTTCGxCCAACCCGTGdTCCAATCCCAgaAAGCGGCCGCGLAGCG
D2-4-78	(-)			LgatatggtctactgCTTCACGAGTCAAGGxCTCCAATCCGTGxTCCAACCAGTTTTgacaagcggagtagttagacc
D2-4d-56h	+			CGCCCGTCAAGGxCTCCAATCCGTGdTCCAACCAGTTTTgacGGgcgCGCGLAGCG
D2-5-46h	+			GCCCGCGTGCTCAACCTTACCAATCTGxCACGCGGGCCGCGLAGCG
D2-5-48h	+			GCCCTGCGxGCTCAACCTTACCAATCTGxCACGCAGGGCCGCGLAGCG
D2-6-54h	-			GCCCTGTCGAGAxGCACCAACCAACCCAATCTGxCTTGACAGGGCCGCGLAGCG
**Name**	**EMSA**	**SPR**		**Sequence (5'- to -3': L = Biotin-dT, x = dDs)**
D3-1-85	(++)			LactccatgatatggtctactgATAGTACTCCxGTTTAACTCTGAxACTTGACGTCCATTCATAgacaagcggagtagttagacc
**D3-2-78**	**(+++) +++**	**K_D = 41 pM**	**specific**	LgatatggtctactgAAGTGTTGTCATCTAxCCTGGCCxTGTGGTACTGTAACGGCTgacaagcggagtagttagacc
**D3-2-59h (AptD3)**	**+++**	**K_D = 57 pM**	**specific**	CCGCTTGTCATCTAxCCTGGCCxTGTGGTACTGTAACGGCTgacaagcggCGCGLAGCG
**Cont-D3-2-59h**	**-**	**K_D = 0.19 μM**		CCGCTTGTCATCTAACCTGGCCATGTGGTACTGTAACGGCTGACAAGCGGCGCGLAGCG
D3-3-78	(+)			LgatatggtctactgGGGCTTGGTCTTGCGTxTGCAGATTAACTTGCGTGCCAGTAAgacaagcggagtagttagacc
**Name**	**EMSA**	**SPR**		**Sequence (5'- to -3': L = Biotin-dT, x = dDs)**
D4-1-78	(+++) +++	K_D = 42 pM	specific	LgatatggtctactgGTCTCAACGGTTGTCAAACGGxTATCACGGCxACACACCTGCGgacaagcggagtagttagacc
D4-1-57h	+++	K_D = 29 pM	specific	CTCCGCTGTCAAACGGxTATCACGGCxACACACCTGCGgaca-gcggagCGCGLAGCG
D4-2-78	(+++)			LgatatggtctactgTCACAxATCGCCGTAAAGxCGAAGAGCTGCGGAATCTAAGGTgacaagcggagtagttagacc
**D4-3-78**	**(+++) +++**	**K_D = 34 pM**	**specific**	LgatatggtctactgGAGGAGACGTAACGCxTATCAAATCxAAACAGCTTAGGGTCCgacaagcggagtagttagacc
**D4-3-57h (AptD4)**	**+++**	**K_D = 30 pM**	**specific**	CgGCGGAGACGTAACGCxTATCAAATCxAAACAGCTTAGGGTCCgCcGCGCGLAGCG
**Cont-D4-3-57h**	**-**	**K_D = 2.0 nM**		CgGCGGAGACGTAACGCATATCAAATCAAAACAGCTTAGGGTCCgCcGCGCGLAGCG
D4-4-78	(+++)			LgatatggtctactgTATAATCCGCxTTCGTCATGTGGxTTGGATCTGGGTCTGGCAgacaagcggagtagttagacc
D4-5-78	(++)			LgatatggtctactgCCCAAxCTTGTCTGTAAGGGxTTGGxTAGGGCTGGCAAAAAAgacaagcggagtagttagacc

In parentheses: EMSA using 2M urea gel
Relative shifted ratio (%)
-: <10%
+: 10-40%
++: 40-60%
+++: >60%

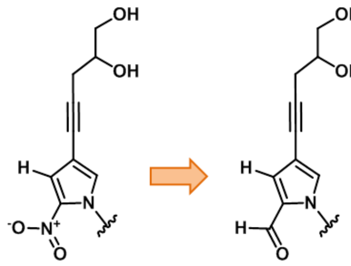

Px   Pa

```
DEN1-NS1      DSGCVINWKGRELKCGSGIFVTNEVHTWTEQYKFQADSPKRLSAAIGKAWEEGVCGIRSA   60
DEN2-NS1      DSGCVVSWKNKELKCGSGIFITDNVHTWTEQYKFQPESPSKLASAIQKAHEEGICGIRSV   60
DEN3-NS1      DMGCVINWKGKELKCGSGIFVTNEVHTWTEQYKFQADSPKRLATAIAGAWENGVCGIRST   60
DEN4-NS1      DMGCVASWSGKELKCGSGIFVVDNVHTWTEQYKFQPESPARLASAILNAHKDGVCGIRST   60
              * *** .*..:*********:.::*********** :** :*::**  * ::*:*****.

DEN1-NS1      TRLENIMWKQISNELNHILLENDMKFTVVVGDVSGILAQGKKMIRPQPMEHKYSWKSWGK   120
DEN2-NS1      TRLENLMWKQITPELNHILSENEVKLTIMTGDIKGIMQAGKRSLRPQPTELKYSWKTWGK   120
DEN3-NS1      TRMENLLWKQIANELNYILWENNIKLTVVVGDTLGVLEQGKRTLTPQPMELKYSWKTWGK   120
DEN4-NS1      TRLENVMWKQITNELNYVLWEGGHDLTVVAGDVKGVLTKGKRALTPPVSDLKYSWKTWGK   120
              **:**::****: ***::* *.  .:*::.**  *::  **: : *   : *****:***

DEN1-NS1      AKIIGADVQNTTFIIDGPNTPECPDNQRAWNIWEVEDYGFGIFTTNIWLKLRDSYTQVCD   180
DEN2-NS1      AKMLSTESHNQTFLIDGPETAECPNTNRAWNSLEVEDYGFGVFTTNIWLKLKEKQDVFCD   180
DEN3-NS1      AKIVTAETQNSSFIIDGPNTPECPSASRAWNVWEVEDYGFGVFTTNIWLKLREVYTQLCD   180
DEN4-NS1      AKIFTPEARNSTFLIDGPDTSECPNERRAWNSLEVEDYGFGMFTTNIWMKFREGSSEVCD   180
              **:.  : :* :*:****:* ***.  ****  ********:******:*:::    .**

DEN1-NS1      HRLMSAAIKDSKAVHADMGYWIESEKNETWKLARASFIEVKTCIWPKSHTLWSNGVLESE   240
DEN2-NS1      SKLMSAAIKDNRAVHADMGYWIESALNDTWKIEKASFIEVKNCHWPKSHTLWSNGVLESE   240
DEN4-NS1      HRLMSAAIKDQKAVHADMGYWIESSKNQTWQIEKASLIEVKTCLWPKTHTLWSNGVLESQ   240
DEN3-NS1      HRLMSAAVKDERAVHADMGYWIESQKNGSWKLEKASLIEVKTCTWPKSHTLWTNGVLESD   240
               :*****:**.:************  * :*:: :**:****.* ***:****:******:

DEN1-NS1      MIIPKIYGGPISQHNYRPGYFTQTAGPWHLGKLELDFDLCEGTTVVVDEHCGNRGPSLRT   300
DEN2-NS1      MIIPKNLAGPVSQHNYRPGYHTQITGPWHLGKLEMDFDFCDGTTVVVTEDCGNRGPSLRT   300
DEN4-NS1      MLIPKSYAGPFSQHNYRQGYATQTVGPWHLGKLEIDFGECPGTTVTIQEDCDHRGPSLRT   300
DEN3-NS1      MIIPKSLAGPISQHNYRPGYHTQTAGPWHLGKLELDFNYCEGTTVVITESCGTRGPSLRT   300
              *:***  .**.****** ** ** .*********:**. * ****.: * *. *******

DEN1-NS1      TTVTGKTIHEWCCRSCTLPPLRFKGEDGCWYGMEIRPVKEKEENLVKSMVSA   352
DEN2-NS1      TTASGKLITEWCCRSCTLPPLRYRGEDGCWYGMEIRPLKEKEENLVNSLVTA   352
DEN4-NS1      TTASGKLVTQWCCRSCTMPPLRFLGEDGCWYGMEIRPLSEKEENMVKSQVTA   352
DEN3-NS1      TTVSGKLIHEWCCRSCTLPPLRYMGEDGCWYGMEIRPISEKEENMVKSLVSA   352
              **.:** : :*******:****: *************:.*****:*:* *:*
```

Amino Acid Sequence Identity (%)

	DEN1-NS1	DEN2-NS1	DEN3-NS1	DEN4-NS1
DEN1-NS1	**100.0**	72.7	79.8	69.3
DEN2-NS1	72.7	**100.0**	73.6	72.7
DEN3-NS1	79.8	73.6	**100.00**	73.9
DEN4-NS1	69.3	72.7	73.9	**100.0**

Supplementary Fig. S1: Sequences of four serotypes of DEN-NS1 proteins used in ExSELEX. The amino acid sequences of each serotype DEN-NS1 protein were obtained from The Native Antigen Company (DEN1-NS: Nauru/Western Pacific/1974; DEN2-NS1: Thailand/16681/84; DEN3-NS1: Sri Lanka D3/H/IMTSSA-SRI/2000/1266; DEN4-NS1: Dominica/814669/1981). The amino acid sequence alignment and identity were analyzed with Clustal Omega (https://www.ebi.ac.uk/Tools/msa/clustalo/).

Monoclonal antibody: Ab#D06

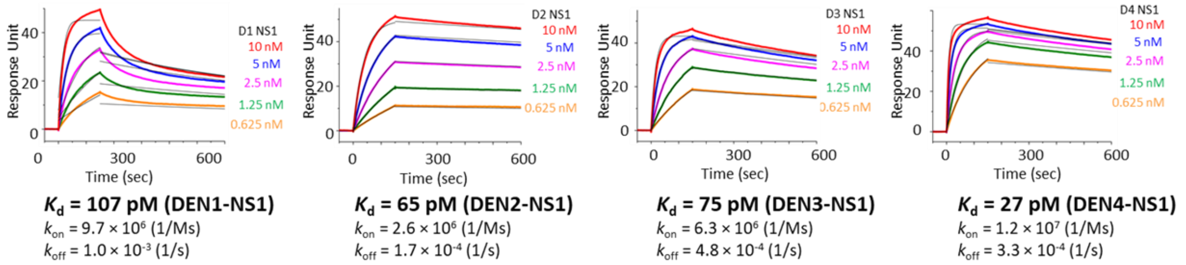

Monoclonal antibody: Ab#D25

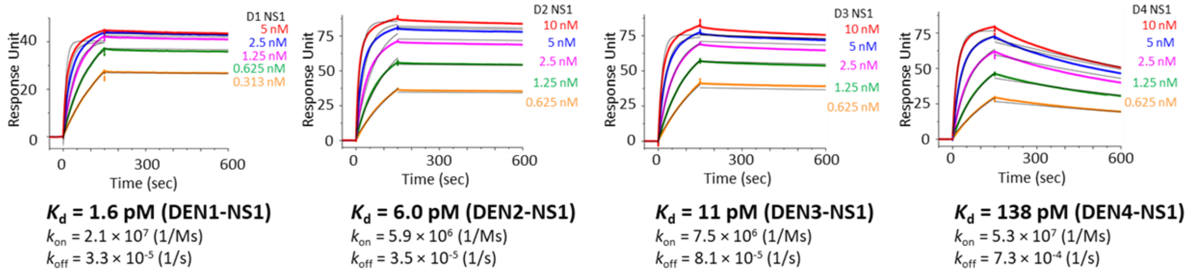

Supplementary Fig. S2: The kinetic binding parameters, dissociation constant (K_D) and association and dissociation rates (k_{on} and k_{off}) of rabbit monoclonal antibodies, Ab#D06 and Ab#D25. These kinetic binding parameters were determined by SPR analyses with various concentrations of each DEN-NS1 serotype.

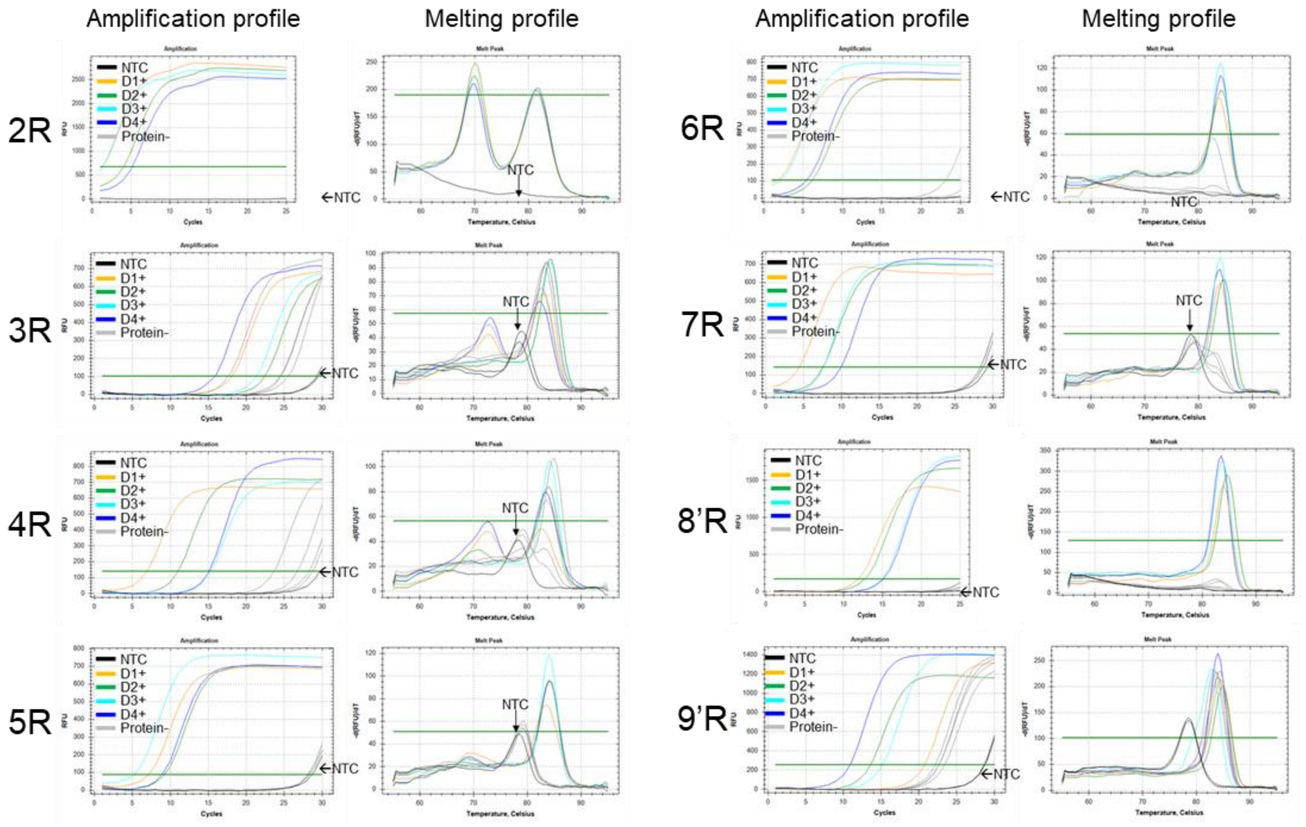

Supplementary Fig. S3: Quantitative PCR analysis of ExSELEX-1. PCR amplification curves and their melting curves in ExSELEX-1 were monitored using a Bio-Rad CFX96 PCR system. For the inputs, small portions of the recovered DNA in each selection round, in the presence of each target (DEN1-NS1 (D1+): orange, DEN2-NS1 (D2+): green, DEN3-NS1 (D3+): cyan, and DEN4-NS1 (D4+): blue) or in the absence of the target (protein-: gray) were used. Amplification cycles for the large-scale PCR (Supplementary Table 2) to prepare the libraries for the next round of selection were determined from the PCR amplification curves.

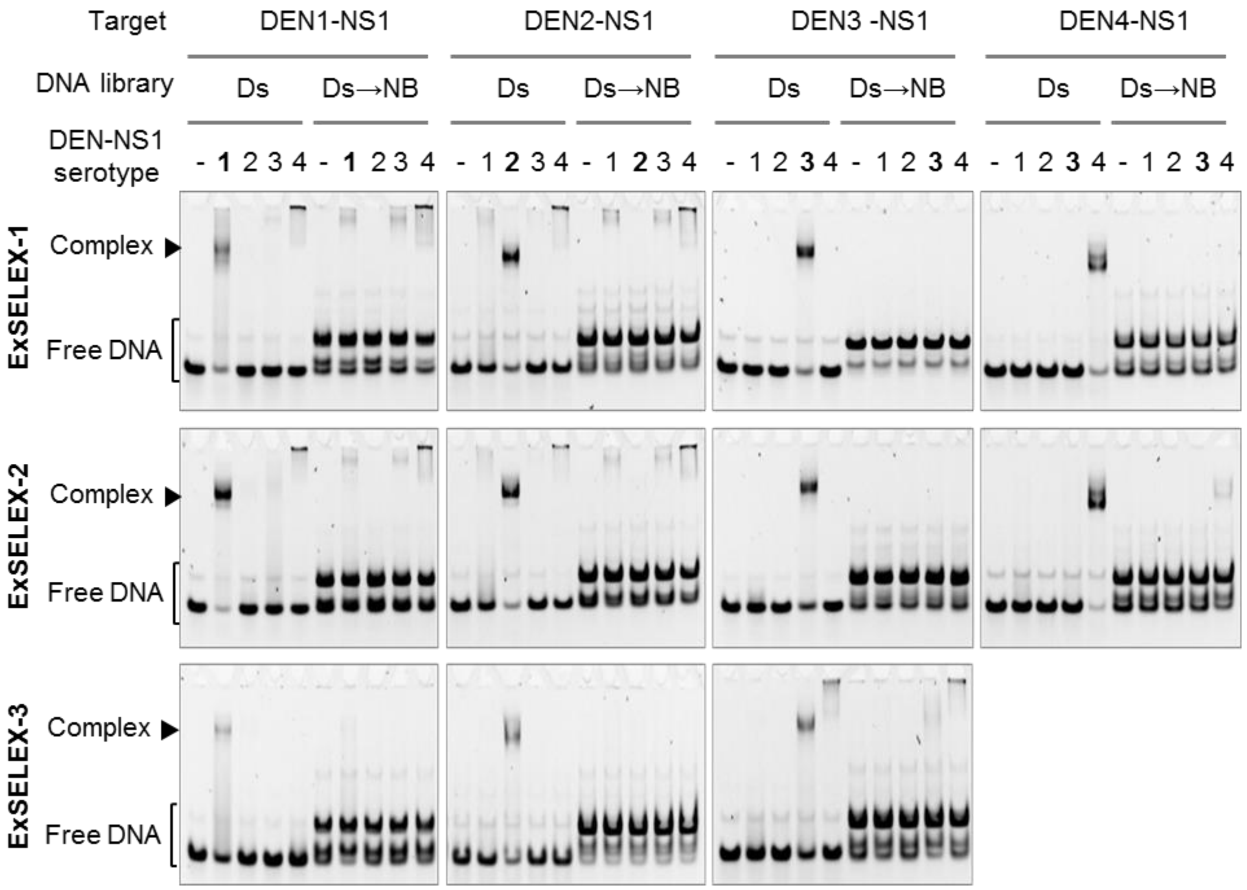

Supplementary Fig. S4: Binding analysis of DNA libraries by electrophoresis gel-mobility shift assays (EMSA). The enriched DNA libraries (50 nM) in the final round of three independent ExSELEX procedures (ExSELEX-1, ExSELEX-2, and ExSELEX-3 targeting each DEN-NS1 protein) were incubated with DEN1-NS1 (NAD1), DEN2-NS1 (NAD2), DEN3-NS1 (NAD3) or DEN4-NS1 (NAD4) (25 nM as the hexamer form; total 150 nM monomeric units) in binding buffer supplemented with 0.05% Nonidet P-40 at 25°C for 30 min, The DNA–NS1 complexes were separated from the free DNA on native 4% acrylamide gels. The DNA band patterns on the gels were detected with a bio-imaging analyzer (LAS4000), after staining the DNA bands with SYBR Gold. To investigate the importance of the Ds bases in the DNA libraries, we prepared the DNA libraries without the Ds bases by replacement PCR, and performed a comparative analysis of the binding patterns (Ds vs. Ds→natural base (NB)).

ExSELEX-Target	Family	Ratio / % (counts)	Random and partial primer regions
ExSELEX-1-D1	D1-1	10.01 (3943)	TGTGAxGTCCTACAATGGACTGGTGTxCTCGG-xATGGCCATTgacaagcggagta
ExSELEX-1-D1		8.79 (3464)	GCAGCTATGCTCAAGATCCCTGGTGTxCTCGG-xATGGGACTTgacaagcggagta
ExSELEX-1-D1		7.39 (2912)	tactgTCCGCTTAGCxAGCTGCCTGGTGTxCTCGG-xATGGTCTGTGCgacaagcggagt
ExSELEX-1-D1		2.63 (1037)	CCGTAACGTCxGGTTCGCTGGTGTxCTCGG-xATGCTGATCCCgacaagcg
ExSELEX-1-D1		2.52 (994)	atgatatggtctactgCACTGGTGTxCTCGG-xATGGGCAGTGACCATATTTCACTGGA
ExSELEX-1-D1		3.75 (1479)	TACTGTCCGCATGTCxGTGCTGGTGTxCTCGG-xATGCTACGTgacaagcggagta
ExSELEX-1-D1		1.68 (663)	ggtctactgGAGAGxATGCGCTGGTGTxCTCGG-xATGCGCTCACTTCATTA
ExSELEX-1-D1		1.57 (617)	ATGGCCTATGTCCCAxGGCTGGTGTxCTCGG-xATGCCAAAGGgacaagcgga
ExSELEX-1-D1		1.54 (608)	CAAATGTCTGAGAAAACTCTGGTGTxCTCGGGxATGAGTTTGgacaagcggagt
ExSELEX-1-D1		1.50 (591)	GCAAGxATCCGAGCCCTTGTCAGCTGGTGTxCTCGG-xAATGGTgacaagcggagtag
ExSELEX-1-D1		1.41 (555)	atggtctactgGGGGGGxGCCTGGTGTxCTCGG-xATGGCTCCCCCAAAACGAT
ExSELEX-1-D1		1.30 (511)	AGACGTTCTGxTCACCACGCTGGTGTxCTCGG-xATGGGTGGTgacaagcggagt
ExSELEX-1-D1	D1-2	10.62 (4185)	AGGAGCGCATGTCGAGATACCAACCx-CCATCCAATCxTTCTTgacaagcgga
ExSELEX-1-D1		2.46 (968)	ctgGCTTGTCGTGCGCGCCAATCxCCCATCCAACCxTCGTGTAGGgacaagcg
ExSELEX-1-D1		2.79 (1097)	CAGCATGTCACTGCGxCCAATCx-ACAGCCAACCAAAGCAAGTgacaagcg
ExSELEX-1-D1		1.65 (650)	atgatatggtctactgTAGGGTGGGTxTGGGAAGGxACTCGTAAACCATGTCAGTGCGga
ExSELEX-1-D1		1.47 (579)	ATAGAATAGGCCCCGTGTTxATCAGACGCATCCxCATTCGGGgacaagcggag
ExSELEX-2-D1	D1-3	54.58 (30778)	tactgACGCCGGGGCCCGTAxTCAGACGTATA-CxCATCAGGGCACATgacaag
ExSELEX-2-D1	D1-4	22.71 (12808)	CCTGCACTGCCTCTGxAGCCCAACCxCCCATCCAATCGCTCAgacaagcggag
ExSELEX-2-D1		11.73 (6615)	TCTAAxGTCATGAGCCCAACCxCCCATCCAATCGCGATTATAgacaagcg
ExSELEX-2-D1		2.14 (1206)	tactgGCCGGxAGTCGCTACCAATCTAC-CCAACCATGCGxCATGCACgacaag
ExSELEX-2-D1		1.75 (988)	CTGCTTTGTCACAGGAGx-CAATCTAG-CCAACCGTCCGCACTGgacaagcgga
ExSELEX-2-D1		1.34 (758)	actgATTGTCCATAxTCGGTGGCAxTGGCAAGGTTxAGGTTATCCGgacaag
ExSELEX-3-D1	D1-5	7.88 (21188)	GCGACxGCGCGTCGATTGxCCAATCTTAGCCAACCCAAAATTgacaagcgga
ExSELEX-3-D1		8.23 (22148)	atggtctactgCAGCTAAGTCCAACCCACGCCAATCxACGCGGCTGCxATGTAgaca
ExSELEX-3-D1	D1-6	7.05 (18969)	ACGAGCTTAGGACCTxGTACCAACCCCCTCCAATCxATTAGGgacaagcggag
ExSELEX-3-D1		2.22 (5985)	GTATGAAGCTxGACAACGGxCCAACCCCCTCCAATCTTAGTTgacaagcgg
ExSELEX-3-D1		4.11 (11054)	GTCCATTGAACTGAAxACCCAATCACCTCCAACCxGTTGAAGgacaagcggag
ExSELEX-3-D1		2.75 (7408)	CTACGGTTGGCGGATxTTACCAACCTCCTCCAATCxTAGTGCgacaagcggag
ExSELEX-3-D1	D1-7	6.10 (16398)	GACGGTTGTTAACGAxGTCCAACCAGTCCCAATCxACAAGTTgacaagc
ExSELEX-3-D1		3.31 (8905)	TCCGTxAAGGTTGTGCACCCAACCAGCTCCAATCGTxGCACAgacaagcggag
ExSELEX-3-D1		3.20 (8613)	CTCTGTTTGTxAGGAGAGCCAACCAGT-CCAATCCTCACAACTgacaagcggag
ExSELEX-3-D1		5.89 (15835)	GGTAGCGCGGGAGGCxGTCCAACCTAT-CCAATCxACAGTCCGgacaagc
ExSELEX-3-D1		3.22 (8664)	ATACGxATTGACAAGGCCxCCAACCAAA-CCAATCCACGGCCGgacaagc
ExSELEX-3-D1		2.64 (7115)	GTCCGCGAAGTCCCCxACCAACCxGCGCCCAATCAAACCAGGgacaagcggag
ExSELEX-3-D1		2.15 (5797)	GGTAGCATGTTTTCTxGCCCAATCTCCCCCAACCxGCGAGAAgacaagcggag
ExSELEX-3-D1		2.11 (5667)	GCGAGCAGGCxATGCGACCCAATCTAG-CCAACCxGTCGCGTAgacaagcggag

Supplementary Fig. S5: Alignment of the random-region DNA sequences obtained by the three ExSELEX procedures, targeting DEN1-NS1. The sequences were obtained by deep sequencing through replacement PCR, by using an intermediate unnatural-base substrate, dPa′TP. Partial constant sequences, flanking the 42-nucleotide random region, are indicated in lower-case letters. The unnatural-base positions, indicated by a red "x", were predicted from the mutation spectra (natural-base composition rates) after replacement PCR. The ratio (%) of each family was calculated from the total read counts (replacement with Pa′) clustered in the same family (shown in parentheses) against the total extracted reads for the analysis (replacement with Pa′, Supplementary Table S4). Several representative family sequences, from D1-1 to D1-7, were chosen for binding analyses by EMSA and SPR (summarized in Supplementary Table S5). The complementary sequences forming two or more consecutive A–T and G–C/T pairs are shown in yellow, and the consensus sequences found among the families are shown in different colors.

ExSELEX-Target	Family	Ratio / % (counts)	Random and partial primer regions
ExSELEX-1-D2	D2-1	34.41 (17433)	gatatggtctactgGTCCGxCTGGGAACAAGxGGCGGGAGGGAxGGGT-GTGGGTGCgacaagcggagtagttagaccg
ExSELEX-1-D2		7.66 (3800)	atggtctactgAGGGAGTAGxGAGGACCAAGxG-C-GGA-GGAxGGCG-GCGGGTGGgacaagcggagtagttagaccg
ExSELEX-1-D2		1.88 (951)	atggtctactgATTATGGCTCxAGTCAAGxGGCAGTTGGGAxGGGTTGTGGGTgacaagcggagtagttagaccgt
ExSELEX-1-D2		17.36 (8797)	ggtctactgTGGGAAGTCAxGGGGGCxTGGTGTAGTCGGxAGACGGGAGTTgacaagcggagtagt
ExSELEX-1-D2		1.13 (570)	ggtctactgACGTCxAGGTTGGAAAGGxACGGCCGTxAATACATGCACAAGgacaagcggagtagt
ExSELEX-1-D2		20.75 (10512)	ggtctactgCTCTAACCAACAGAGGCCAGxCCAATCTTA-CCAACCxCCCTGgacaagcggagtagt
ExSELEX-1-D2		2.85 (1442)	ggtctactgAGTGTCGACGGGGAGxCCAATCCGC-CCAACCxCCCTCCCCTAgacaagcggagtagt
ExSELEX-1-D2		3.54 (1794)	ggtctactgCTACGTGAAGxTCCAATCTCTACCAACCTGTxTCACGAxAGTgacaagcggagtagt
ExSELEX-1-D2		2.79 (1413)	ggtctactgATAGCCTGTCCGCTTxGTCGCCCAACCCGTGxTCCAATCCAAgacaagcggagtagt
ExSELEX-1-D2		1.40 (709)	ggtctactgAAAGGCGCGTxGTAACTGTCCAATCCGCGxTCCAACCACAGAgacaagcggagtagt
ExSELEX-2-D2	D2-2	23.26 (15556)	ggtctactgGAATAACAAGTCCGTGCxGTCGCCAATCCGTGxTCCAACCCCgacaagcggagtagttagaccg
ExSELEX-2-D2	D2-3	21.67 (14490)	ggtctactgGTCTACAxGCAACGCTTTCGxCCAACCCGTGxTCCAATCCCAgacaagcggagtagt
ExSELEX-2-D2	D2-4	11.97 (8003)	ggtctactgCTTCACGAGTCAAGGxCTCCAATCCGTGxTCCAACCAGTTTTgacaagcggagtagt
ExSELEX-2-D2		6.97 (7781)	ggtctactgACCGCGAAAGTAGGCxTCAACCCGTGxTCCAATCCGCGCCGAgacaagcggagtagt
ExSELEX-2-D2		10.59 (7079)	ggtctactgGCGCTCGGCTCGCGCxTCAATCCGTGxTCCAACCCCGCGCGAgacaagcggagtagt
ExSELEX-2-D2	D2-5	11.63 (5530)	ggtctactgTGGCTGGGCCAxGCGTGCTCAACCTTACCAATCTGxCACGCGgacaagcggagtagt
ExSELEX-2-D2	D2-6	8.27 (4661)	ggtctactgTAGATxTTGTCGAGAxGCACCAACCAACCCAATCTGxCTTGAgacaagcggagtagt
ExSELEX-2-D2		4.16 (2785)	ggtctactgACCCGTCGACCTCTCACCCAACCATCCCAATCxAGCATAAGAgacaagcggagtagt
ExSELEX-3-D2	D2-1	65.94 (193819)	tatggtctactgGTCCGxCTGGGAACAAGxGGCGGGAGGGAxGGGTGTGGGTGCgacaagcggagtagttagaccg
ExSELEX-3-D2		7.99 (23478)	ggtctactgAGACGCGCAGGACTAxGACCAATCTTACCAACCAxCTCAAGGgacaagcggagtagt
ExSELEX-3-D2		2.96 (8712)	ggtctactgCCCGAxTTGCCTGCCCCxCCAACCAGCCCAATCCATGGGGCGgacaagcggagtagt
ExSELEX-3-D2		2.84 (8354)	ggtctactgCCTTGxTATGTCTTCTACCAACCAACCCAATCTAxGAAGACAgacaagcggagtagt
ExSELEX-3-D2		2.65 (7781)	ggtctactgCTGGCxGGGGCGAGGGCCAACCAGACCAATCCCxCGAGACCAgacaagcggagtagt

Supplementary Fig. S6: Alignment of the random-region DNA sequences obtained by the three ExSELEX procedures, targeting DEN2-NS1. The sequences were obtained by deep sequencing through replacement PCR, by using an intermediate unnatural-base substrate, dPa′TP. Partial constant sequences, flanking the 42-nucleotide random region, are indicated in lower-case letters. The unnatural-base positions, shown by the red "x", were predicted from the mutation spectra (natural-base composition rates) after replacement PCR. The ratio (%) of each family was calculated from the total counts (replacement with Pa′) clustered in the same family against the total extracted reads for the analysis (replacement with Pa′, Supplementary Table S4). Several representative family sequences, from D2-1 to D2-6, were chosen for binding analyses by EMSA and SPR (summarized in Supplementary Table S5). The complementary sequences forming two or more consecutive A–T and G–C/T pairs are shown in yellow, and the consensus sequences found among the families are shown in different colors.

ExSELEX-Target	Family	Ratio / % (counts)	Random and partial primer regions
ExSELEX-1-D3	D3-1	89.07 (40625)	gtctactgATAGTACTCCxGTTTAACTCTGAxACTTGACGTCCATTCATAgacaagcggagtagttagac
ExSELEX-1-D3		3.09 (1409)	gtctactgCTGAAGCTCCGTGCGxCCCCC-GCGGTTTGTT-AAAxCCCTTTAgacaagcggagtagttag
ExSELEX-1-D3		2.64 (1202)	atatggtctactgCTTGCxCCCCCCGCGGTTTGTTTAATGGxAGAATTCTGTTAAgacaagcgga
ExSELEX-1-D3		2.00 (911)	atatggtctactgGTCGCTTTGCxCCCCGTCCGGTGACTGAAAATAxCACGTCAGgacaagcggagtagt
ExSELEX-2-D3	D3-2	81.23 (52430)	atggtctactgAAGTGTTGTCATCTAxCCTGGCCxTGTGGTACTGTAACGGCTgacaagcggagtagtta
ExSELEX-2-D3	D3-3	15.56 (10040)	atggtctactgGGGCTxGGTCTTGCGTxTGCAGATTAACTTGCGTGCCAGTAAgacaagcggagtagtta
ExSELEX-3-D3	D3-1	34.13 (72313)	gtctactgATAGTACTCCxGTTTAACTCTGAxACTTGACGTCCATTCATAgacaagcggagtagttagac
ExSELEX-3-D3		27.21 (57653)	atggtctactgTACCACTTGCAATGGACGCGxCGTATGGTGGxTCGGGGAATGgacaagcggagtagtta
ExSELEX-3-D3		8.17 (17312)	tatggtctactgGTGTAATCCGGTTGTGAGACGGCGGAATGxATTTGGAAAGGCgacaagcggagtagt
ExSELEX-3-D3		8.07 (17097)	atatggtctactgCGCGCTTGGGxGACGAATTTGTACAGCGTATATCCAxCGACGgacaagcggagtagt
ExSELEX-3-D3		4.85 (10283)	gatatggtctactgCTCTGTGCCGTCGAGxGACCCTTAGGTTCxCAGCTGCTAACCgacaagcggagt
ExSELEX-3-D3		2.83 (6003)	gatatggtctactgCGTGGTCGAGxGGGCGTGTTTAAACGCATCACAGCCGTAGCCgacaagc
ExSELEX-3-D3		3.14 (6662)	tatggtctactgACACCGTCTxTGTATGTCGCATTCCTGACTCTAxCCCCGACAgacaagcggagtagtt

Supplementary Fig. S7: Alignment of the random-region DNA sequences obtained by the three ExSELEX procedures, targeting DEN3-NS1. The sequences were obtained by deep sequencing through replacement PCR, by using an intermediate unnatural-base substrate, dPa′TP. Partial constant sequences, flanking the 42-nucleotide random region, are indicated in lower-case letters. The unnatural-base positions, indicated by the red "x", were predicted from the mutation spectra (natural-base composition rates) after replacement PCR. The ratio (%) of each family was calculated from the total counts (replacement with Pa′) clustered in the same family against the total extracted reads for the analysis (replacement with Pa′, Supplementary Table S4). Several representative family sequences, from D3-1 to D3-3, were chosen for binding analyses by EMSA and SPR (summarized in Supplementary Table S5). The complementary sequences forming two or more consecutive A–T and G–C/T pairs are shown in yellow, and the consensus sequences found among the families are shown in different colors.

ExSELEX-Target	Family	Ratio / % (counts)	Random and partial primer regions
ExSELEX-1-D4	D4-1	39.19(15319)	tactgTCTCAACGGTTGTCAAACGGxTATCACGGCxACACACCTGCGgacaagcggagtagt
ExSELEX-1-D4		13.74(5371)	GAAAACAGCTTTATCATATAAACGCxTATCACGgacaagcggagtagt
ExSELEX-1-D4		2.38(929)	actgCGGTAxCGCTAAAGGACGGxTATCAAATTA-AAACACCTCCTTgacaagcggagtagt
ExSELEX-1-D4		9.18(3590)	actgTAAGAACAGCGCTGTGAACGCxTATCAAATC-xAAACAGCTTCgacaagcggagtagt
ExSELEX-1-D4		2.88(1125)	tactgTCCTCTAAAGCTGTCCGCGCxTATCAAAGGAxAAACAGCCGAgacaagcggagtagt
ExSELEX-1-D4		4.40(1719)	actgTGGGGxGCGTGAGCTTGGCAAGGGxTTGGxTAGGGCTGGCAAgacaagcggagtagt
ExSELEX-1-D4	D4-2	23.92(9350)	ggtctactgTCACAxATCGCCGTAAAGxCGAAGAGCTGCGGAATCTAAGGTgacaagcggagt
ExSELEX-1-D4		1.29(506)	gtctactgGTCCCTCGTCCAACCGTGCCxACTCTCACTxGAGACCCAATCgacaagcggagt
ExSELEX-2-D4	D4-3	21.96 (9619)	ggtctactgGAGGAGACGTAACGCx-TATCAAATCxAAA-CAGCTTAGGGTCCgacaagcggagtag
ExSELEX-2-D4		17.04 (7461)	actgTGGCGCGAGGGAATCxACGCx-TATCAAATAxAAA-CAGCTAATgacaagcggagtag
ExSELEX-2-D4		8.60 (3766)	actgTCGCACGTTAAACGCxACGGx-TATCAAATCxAAA-CACCTGAGgacaagcggagtag
ExSELEX-2-D4		1.78 (780)	actgGGCACCCATTGTCTCAACGCxATATCAAATCxAAA-CAGCTAGCgacaagcggagtag
ExSELEX-2-D4		1.43 (627)	actgGGCACCCATTGTCTCAACGCx-TATCAAATCxAAAACAGCTGAgacaagcggagtag
ExSELEX-2-D4	D4-4	23.35 (10228)	tactgTATAATCCGCxTTCGTCATGTGGxTTGGATCT-GGGT-CTGGCAgacaagcggagtagt
ExSELEX-2-D4		7.28 (3189)	ctactgCATAGCGGCAxCGGTCGGTGGGxTTGGCxGTGGGGC-TGGCACgacaagcggagtagt
ExSELEX-2-D4	D4-5	3.50 (1535)	gtctactgCCCAAxCTTGTCTGTAAGGGxTTGG--xTAGGGC-TGGCAAAAAgacaagcggag
ExSELEX-2-D4		2.09 (915)	gtctactgGTCTGTGGGGxCTTGAAGGGxTTGG--xTAGGGC-TGGCAAACAAgacaagcggag
ExSELEX-2-D4		1.51 (660)	tctactgGTTTTxGGTTAGTTCTTTGGGxTTGGCAx CGGGCCTGGCGTGgacaagcggagtag

Supplementary Fig. S8: Alignment of the random-region DNA sequences obtained by the three ExSELEX procedures, targeting DEN4-NS1. The sequences were obtained by deep sequencing through replacement PCR, by using an intermediate unnatural-base substrate, dPa′TP. Partial constant sequences, flanking the 42-nucleotide random region, are indicated in lower-case letters. The unnatural-base positions, indicated by the red "x", were predicted from the mutation spectra (natural-base composition rates) after replacement PCR. The ratio (%) of each family was calculated from the total counts (replacement with Pa′) clustered in the same family against the total extracted reads for the analysis (replacement with Pa′, Supplementary Table S4). Several representative family sequences, from D4-1 to D4-5, were chosen for binding analyses by EMSA and SPR (summarized in Supplementary Table S5). The complementary sequences forming two or more consecutive A–T and G–C/T pairs are shown in yellow, and the consensus sequences found among the families are shown in different colors.

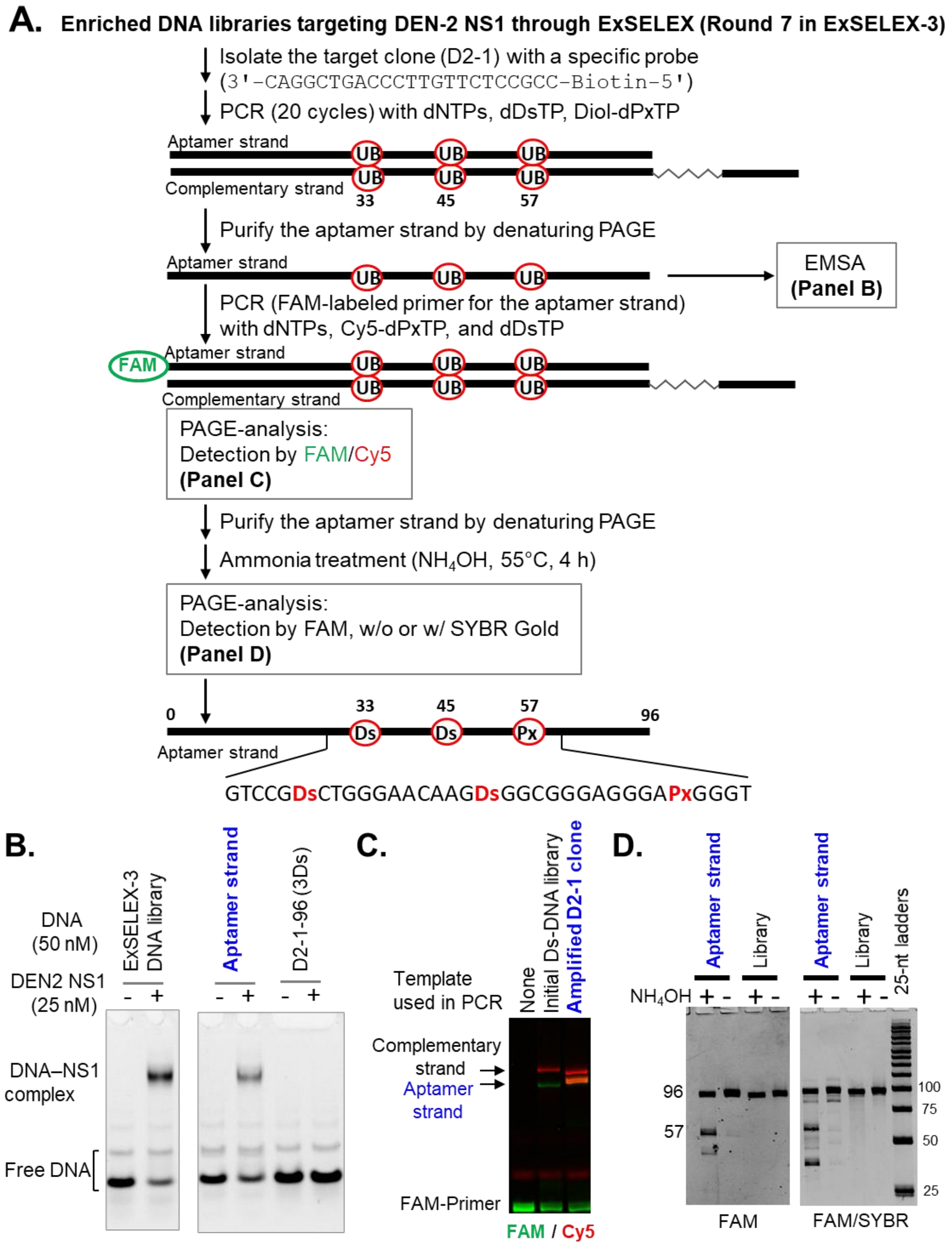

Supplementary Fig. S9: Confirmation of the presence of diol-Px in the selected clone family, D2-1. (**A**) Schematic illustration of the experiment flow to identify a diol-Px position in the family D2-1 isolate. (**B**) Gel mobility shift patterns support the binding of the aptamer strand to the target DEN2-NS1, while the D2-1-96(3Ds), in which the predicted unnatural base positions are all Ds (see Supplementary Table S5), did not bind to the target. (**C**) The Cy5-labelled DNA band patterns, shown in red on the gel image, indicate that the aptamer strand that was PCR-amplified from the isolated clone should contain a Px base, while PCR using an initial Ds-DNA library only produced the aptamer strand without Px bases (no Cy5 incorporation) and the complementary strand with Px bases (Cy5 incorporation). (**D**) The DNA band patterns on the gel images indicate that the aptamer strand was cleaved during a four-hour incubation at 55°C under basic conditions (concentrated ammonia), due to the presence of the Px base, but not the Ds base, at the specific position corresponding to the predicted third Ds base (position 57 of the aptamer strand).

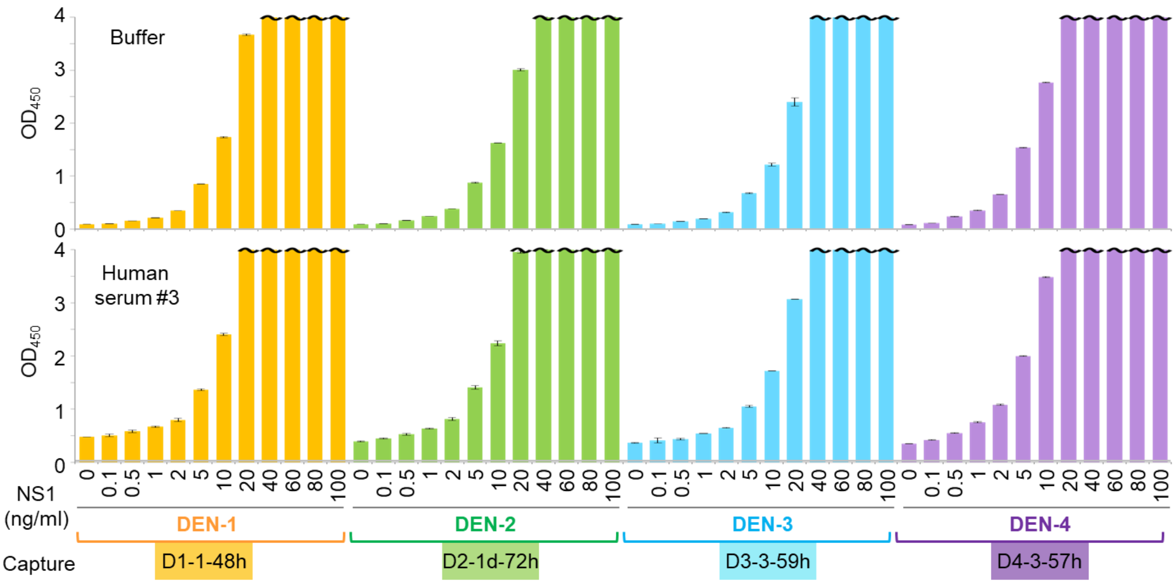

10 µl in assays	DEN1-NS1 (ng/ml)		DEN2-NS1 (ng/ml)		DEN3-NS1 (ng/ml)		DEN4-NS1 (ng/ml)	
	Buffer	HS	Buffer	HS	Buffer	HS	Buffer	HS
LOD (blank+ 3δ)	1.60	2.22	1.86	1.99	2.36	3.31	1.19	1.14
LOQ (blank+ 10δ)	4.77	6.97	5.84	6.91	7.34	11.06	3.92	4.10

Supplementary Fig. S10: Limit of detection (LOD) and limit of quantification (LOQ) targeting each dengue serotype NS1 by a sandwich-type ELISA. UB-DNA aptamers and an anti-DEN-NS1 monoclonal antibody (Ab# D06) were used as capture agents and the primary detector agent, respectively. For the target binding process, 10 µl of serially diluted NS1 (0 to 100 ng/ml) was used in buffer with and without control human serum (PD0-1, 10%) in each 100-µl binding solution. The sample size is two per each combination set, and the data are from two independent experiments. The error bars represent one average deviation. The bars with wavy lines indicate that the signal in at least one of the two sample wells was saturated (OD_{450} > 4.000).

A

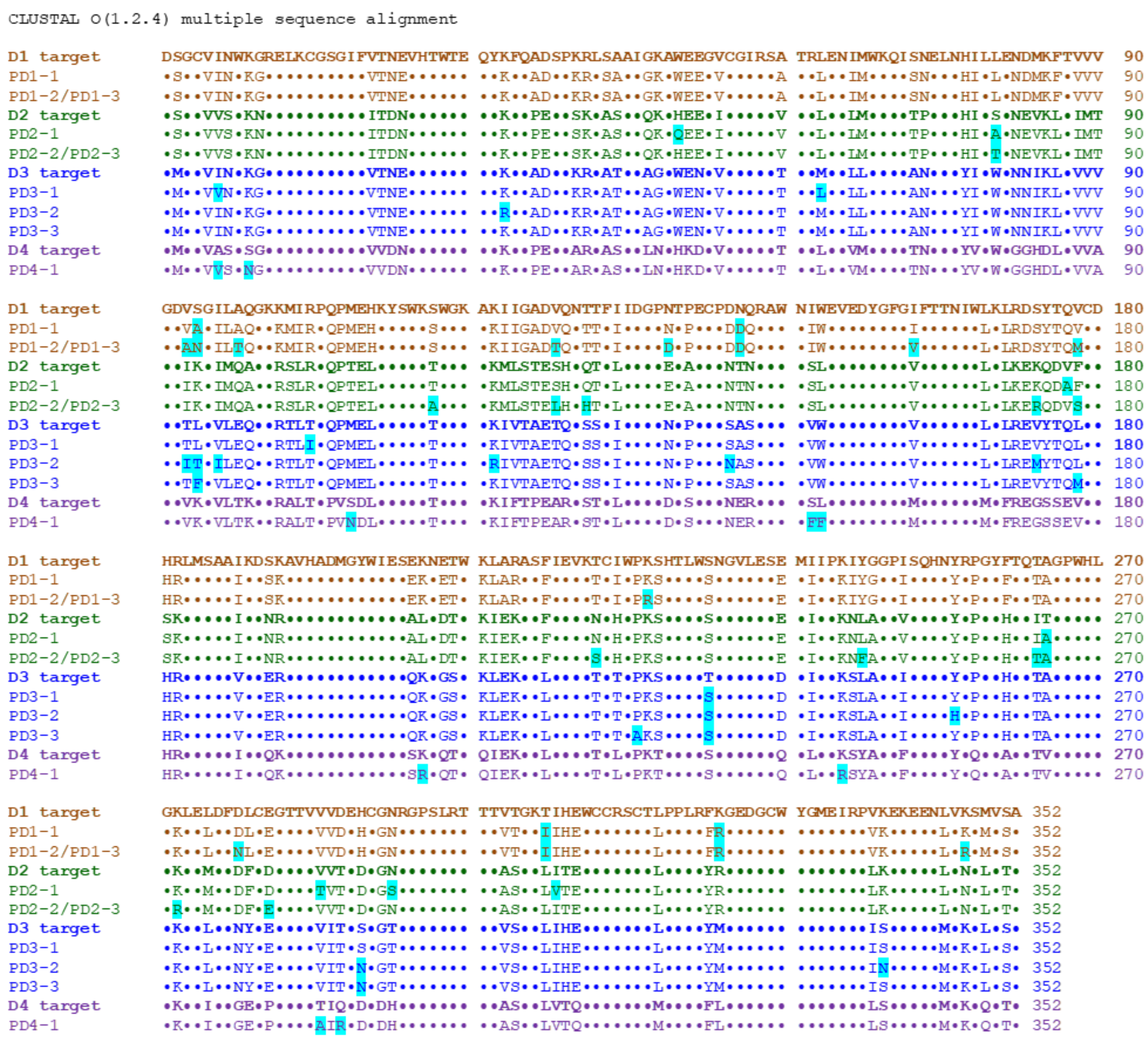

B

Mutated Amino Acids (in 352 Amino Acids)	D1 target	PD1-1	PD1-2 PD1-3	D2 target	PD2-1	PD2-2 PD2-3	D3 target	PD3-1	PD3-2	PD3-3	D4 target	PD4-1
		4	13		7	12		4	11	5		9
D1_target	100.0	98.9	96.3	72.7	72.2	73.3	79.8	80.1	79.6	79.8	69.3	68.8
D2_target	72.7	73.0	72.7	100.0	98.0	96.6	73.6	74.4	73.9	73.6	72.7	72.2
D3_target	79.8	79.8	79.8	73.6	73.3	73.6	100.0	98.9	96.9	98.6	73.9	73.3
D4_target	69.3	69.3	68.8	72.7	73.0	72.4	73.9	74.2	73.0	73.9	100.0	97.4

Supplementary Fig. S11: Differences in the amino acid sequences of DEN-NS1 proteins in the clinical samples. (**A**) Alignment of the amino acid sequences of DEN-NS1 proteins in clinical samples and each recombinant DEN-NS1 protein used in aptamer generation as the target. The common amino acids in the sequences are denoted with asterisks. Each serotype is colored: DEN1-NS1 is dark orange, DEN2-NS1 is green, DEN3-NS1 is blue, and DEN4-NS1 is purple. Amino acids that are different from those in each targeted serotype NS1 protein are highlighted in light blue. (**B**) Summary of the sequence identity of the NS1 sequences, with mutation numbers, compared with each target NS1 protein sequence. The samples in which we successfully detected NS1 with the ELISA format, using the specific UB-DNA aptamers, are highlighted with each serotype's color.

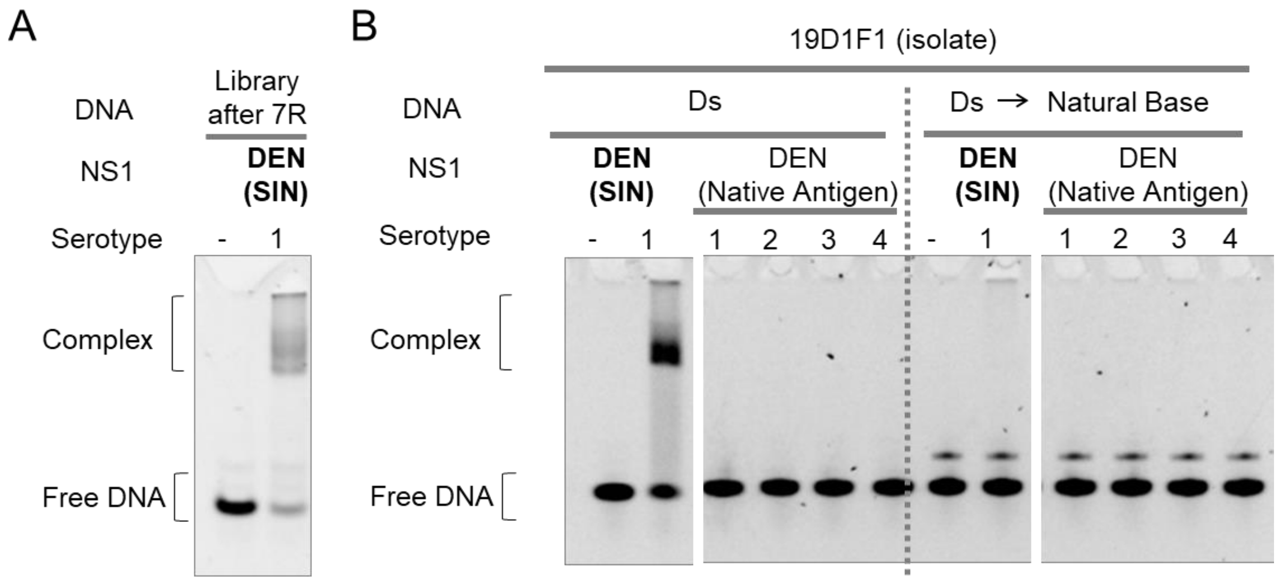

Supplementary Fig. S12: Binding analysis of the enriched DNA library and 19D1F1 to DEN-NS1 variant 2 by EMSA. The enriched DNA library in the final round of ExSELEX-4 (A) and the isolated clone 19D1F1 (B) were incubated with DEN1-NS1 variant 2 (SIN), DEN1-NS1, DEN2-NS1, DEN3-NS1 and DEN4-NS1 from The Native Antigen Company, in binding buffer supplemented with 0.05% Nonidet P-40, at 25°C for 30 min. The DNA–NS1 complexes were separated from the free DNA on a native 4% acrylamide gel (A) and a 4% acrylamide gel in the presence of 2 M urea (B). DNA: 50 nM, DEN-NS1: 25 nM as the hexamer form; total 150 nM monomeric units.

A

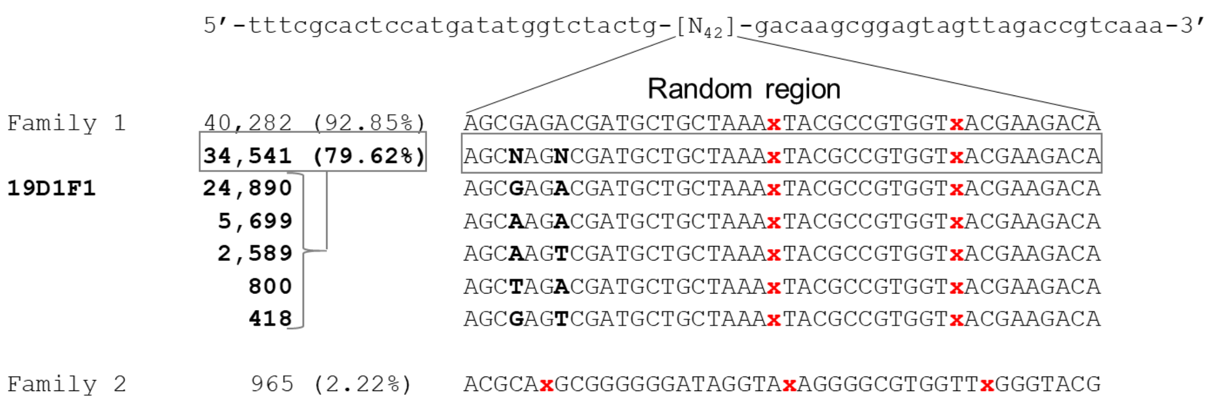

B

DNA	Preparation	ELISA detection: OD_{450} for recombinant DEN-NS1			SPR: K_D	Sequence (5'- to -3' : I = Biotin, L = Biotin-dT, x = dDs, y = Diol-dPx, N = natural bases)
		None	variant 2	variant 1 (NAD1)	variant 2	
19D1F1(isolate)	PCR	0.117	**>4.000**	0.116	**9.1 pM**	ItttcgcactccatgatatggtctactgAGCGAGACGATGCTGCTAAAxTACGCCGTGGTxACGAAGACAgacaagcggagtagttagaccgtgaaa
19D1F1:Ds→NB		0.105	**0.391**	0.101	N.D.	ItttcgcactccatgatatggtctactgAGCGAGACGATGCTGCTAAANTACGCCGTGGTNACGAAGACAgacaagcggagtagttagaccgtgaaa
19D1F1-1	Chemical synthesis	0.104	0.119	0.099	N.A.	CGGCCGATGCTGCTAAAxTACGCCGTGGTxACGAAGACAgacaagcggagtagttagaccGGCCGCGCGLAGCG
19D1F1-2		0.102	0.101	0.096	N.A.	GCGCCAAAxTACGCCGTGGTxACGAAGACAgacaagcggagtagttGGCGCCGCGLAGCG
19D1F1-3 **(AptD1b)**		0.078	**3.592**	0.078	**27 pM**	gcactccatgatatggtctactgAGCGAGACGATGCTGCTAAAxTACGCCGTGGTxACGAAGACAgacaagcggagt gt CGCGLAGCG
19D1F1-4		0.079	0.080	0.079	N.A.	gcactcc gtctactgAGCGAGACGATGCTGCTAAAxTACGCCGTGGTxACGAAGAC ggagt gt CGCGLAGCG
19D1F1-5		0.079	0.083	0.078	N.A.	gcactcc g ctactgAGCGAGACGATGCTGCTAAAxTACGCCGTGGTxACGAAGACAg cggagt gt CGCGLAGCG

C

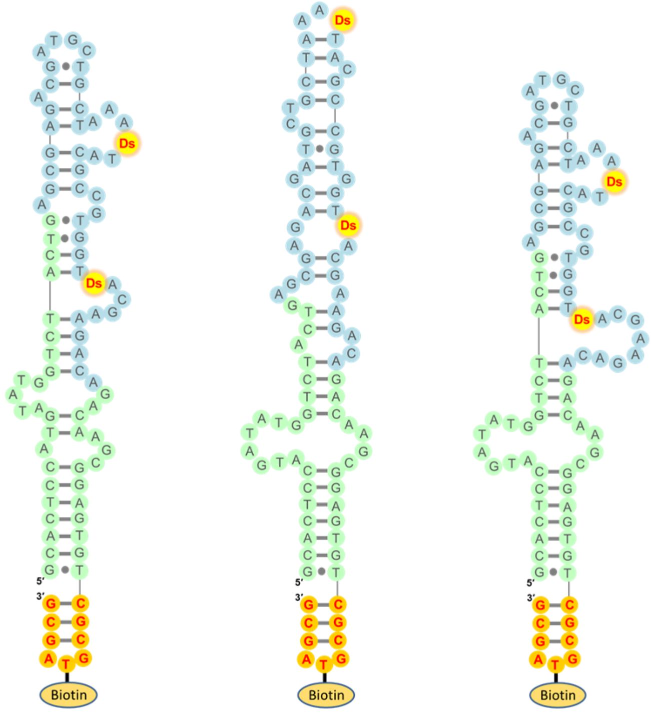

Supplementary Fig. S13: The UB-DNA aptamer generation targeting DEN1-NS1 variant 2. (A) Alignment of the random-region DNA sequences obtained by ExSELEX-4 (Supplementary Table S3). The unnatural-base positions, indicated by "x" in red, were predicted from the mutation spectra (natural-base composition rates) after replacement PCR. The ratio (%) of each family was calculated from the total counts categorized in the same family against the total extracted reads for the analysis. (B) DNA aptamer candidates targeting DEN1-NS1 variant 2. 19D1F1 variants, used for the binding analyses (ELISA and SPR) against the DEN1-NS1 variant 2 are summarized. (C) Possible secondary structures of Apt1Db (19D1F1-3), predicted from the binding activities of its variants. The description of each nucleotide is similar to that in Figure 2.

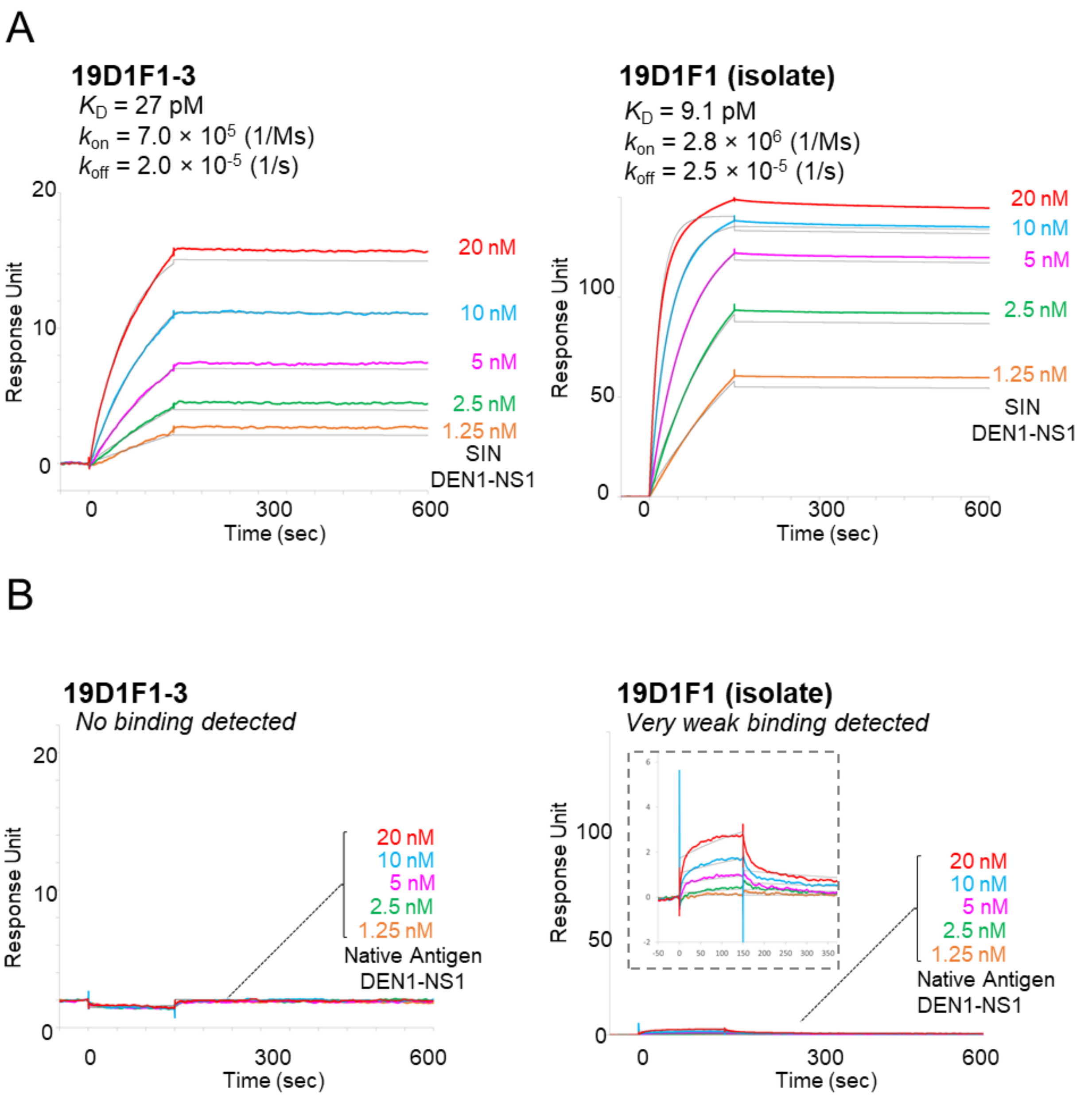

Supplementary Fig. S14: Binding analysis of UB-DNA aptamers, 19D1F1-3 (AptD1b) and 19D1F1 (isolate), to each target by a Biacore T200 SPR system at 25°C. The SPR analysis was performed using the DEN1-NS1 variant 2 (SIN DEN1-NS1) (A) and the DEN1-NS1 from The Native Antigen Company (NAD1) (B). Running buffer: 20 mM Tris-HCl (pH 7.5), 150 mM NaCl, 1 mM $MgCl_2$, 2.7 mM KCl, and 0.05% Tween 20. Flow rate: 30 µl/min. Injection (association) time: 150 sec. Dissociation time: 600 sec (general) or 1,200 sec for determination of kinetic parameters. The kinetic parameters, association rates (k_{on}), dissociation rates (k_{off}), and dissociation constants (K_D), were determined through 1:1 global curve fitting with the BIAevaluation software version 3.0, by using the double-reference subtraction method. Representative association and dissociation curves with fitting (gray lines) are shown. Regeneration was performed with a 5-sec injection of 50 mM NaOH, followed by a 10-min equilibration with running buffer.

```
NAD1                            DSGCVINWKGRELKCGSGIFVTNEVHTWTEQYKFQADSPKRLSAAIGKAWEEGVCGIRSA 60
PD1-1/4/5/6                     ------------------------------------------------------------
PD1-7                           ------------------------------------------------------------
PD1-8                           ------------------------------------------------------------
PD1-9                           ------------------------------------------------------------
PD1-10                          ------------------------------------------------------------
PD1-11/12                       ------------------------------------------------------------
PD1-2/3/13/14/15/16/17/18/19/20 ------------------------------------------------------------
PD1-21                          ------------------------------------------------------------
PD1-22                          ------------------------------------------------------------
NAD1                            TRLENIMWKQISNELNHILLENDMKFTVVVGDVSGILAQGKKMIRPQPMEHKYSWKSWGK 120

PD1-1/4/5/6                     T------------------------------DVA---A----------------------
PD1-7                           T------------------------------DVT---A----------------------
PD1-8                           T------------------------------DVA---A----------------------
PD1-9                           T------------------------------DVA---A----------------------
PD1-10                          T------------------------------NVA---A----------------------
PD1-11/12                       T------------------------------DAN---T----------------------
PD1-2/3/13/14/15/16/17/18/19/20 T------------------------------DAN---T----------------------
PD1-21                          A------------------------------DAN---T----------------------
PD1-22                          T------------------------------DAN---T----------------------
NAD1                            AKIIGADVQNTTFIIDGPNTPECPDNQRAWNIWEVEDYGFGIFTTNIWLKLRDSYTQVCD 180
PD1-1/4/5/6                     ------DV----------N------D---------------I---------------V--
PD1-7                           ------DV----------N------D---------------I---------------V--
PD1-8                           ------DV----------N------D---------------I---------------V--
PD1-9                           ------DV----------N------D---------------I---------------V--
PD1-10                          ------DV----------N------D---------------I---------------V--
PD1-11/12                       ------DT----------D------D---------------V---------------V--
PD1-2/3/13/14/15/16/17/18/19/20 ------DT----------D------D---------------V---------------M--
PD1-21                          ------DT----------D------D---------------V---------------M--
PD1-22                          ------ET----------D------D---------------V---------------M--
NAD1                            HRLMSAAIKDSKAVHADMGYWIESEKNETWKLARASFIEVKTCIWPKSHTLWSNGVLESE 240
PD1-1/4/5/6                     ----------------------------------------------K-------------
PD1-7                           ----------------------------------------------K-------------
PD1-8                           ----------------------------------------------K-------------
PD1-9                           ----------------------------------------------K-------------
PD1-10                          ----------------------------------------------K-------------
PD1-11/12                       ----------------------------------------------R-------------
PD1-2/3/13/14/15/16/17/18/19/20 ----------------------------------------------R-------------
PD1-21                          ----------------------------------------------R-------------
PD1-22                          ----------------------------------------------R-------------
NAD1                            MIIPKIYGGPISQHNYRPGYFTQTAGPWHLGKLELDFDLCEGTTVVVDEHCGNRGPSLRT 300
PD1-1/4/5/6                     -I-----------------------------------D----------------------
PD1-7                           -I-----------------------------------D----------------------
PD1-8                           -V-----------------------------------D----------------------
PD1-9                           -I-----------------------------------N----------------------
PD1-10                          -I-----------------------------------D----------------------
PD1-11/12                       -I-----------------------------------N----------------------
PD1-2/3/13/14/15/16/17/18/19/20 -I-----------------------------------N----------------------
PD1-21                          -I-----------------------------------N----------------------
PD1-22                          -I-----------------------------------N----------------------
NAD1                            TTVTGKTIHEWCCRSCTLPPLRFKGEDGCWYGMEIRPVKEKEENLVKSMVSA 352
PD1-1/4/5/6                     ------I---------------FR----------------------K-----
PD1-7                           ------I---------------FR----------------------K-----
PD1-8                           ------I---------------FR----------------------K-----
PD1-9                           ------I---------------FR----------------------K-----
PD1-10                          ------I---------------YR----------------------K-----
PD1-11/12                       ------I---------------FR----------------------R-----
PD1-2/3/13/14/15/16/17/18/19/20 ------I---------------FR----------------------R-----
PD1-21                          ------I---------------FR----------------------R-----
PD1-22                          ------I---------------FR----------------------R-----
```

Supplementary Fig. S15: Differences in the amino acid sequences of DEN1-NS1 proteins in the clinical samples, PD1-1 through PD1-22. Alignment of the amino acid sequences of DEN1-NS1 proteins in clinical samples and the original DEN1-NS1 protein (NAD1) used in AptD1 aptamer generation as the target. For AptD1b generation, we used the prepared recombinant DEN1-NS1 from PD1-2 and the clinical sample PD1-13. The common amino acids in the sequences are represented by hyphens.

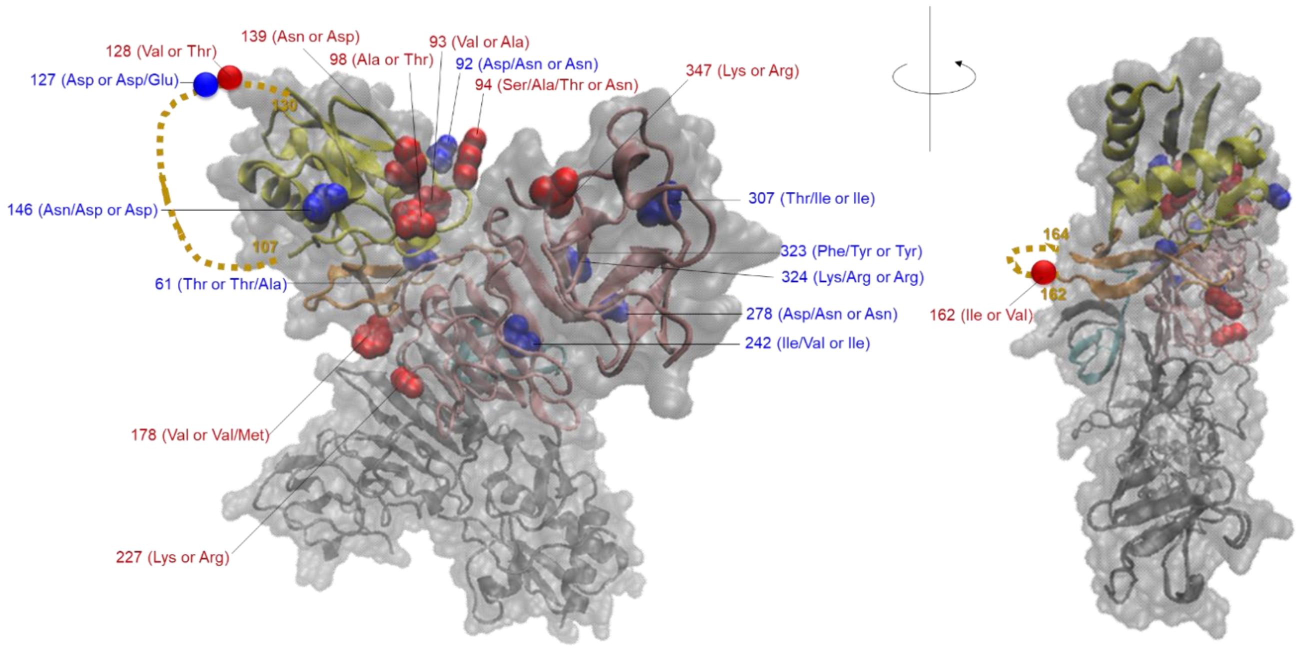

Supplementary Fig. S16: NS1 sequence variations of dengue serotype 1 clinical samples. The amino acids that differed from those in each target dengue NS1 protein from The Native Antigen Company were mapped onto the tertiary structure of the dengue NS1 dimer (PDB: 4O6B), with one subunit in gray and the other colored by domains (cyan, β roll; yellow/orange, wing/connector subdomain; pink, β ladder). The amino acid variations found only in variant 1 (the sequences of The Native Antigen Company and PD1-1 to PD1-10) or variant 2 (PD1-11 to PD1-22) are indicated in blue, while those in PD1-1 to PD1-10 and PD1-11 to PD1-22, which might include critical amino acids for our AptD1 and AptD1b aptamers' binding, are indicated in red. Since the amino acid residue at position 162 would be located within the NS1 hexamer, this residue would be not critical for the specificity of the AptD1 and AptD1b aptamers.

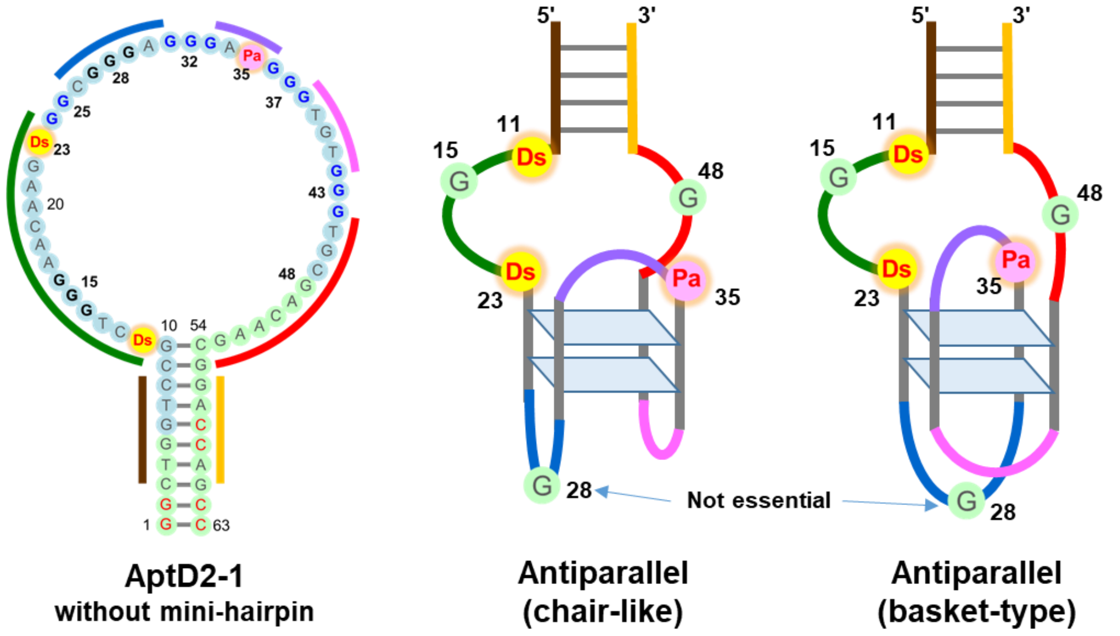

Supplementary Fig. S17: Possible topologies of the G-quadruplex of AptD2-1. The G-to-A scanning experiments indicate the importance of G15 and G48, as well as G25, G32, G37, and G43 in the G-tracts (Fig. 4).
